# STING agonist delivery by tumour-penetrating PEG-lipid nanodiscs primes robust anticancer immunity

**DOI:** 10.1038/s41563-022-01251-z

**Published:** 2022-05-23

**Authors:** Eric L. Dane, Alexis Belessiotis-Richards, Coralie Backlund, Jianing Wang, Kousuke Hidaka, Lauren E. Milling, Sachin Bhagchandani, Mariane B. Melo, Shengwei Wu, Na Li, Nathan Donahue, Kaiyuan Ni, Leyuan Ma, Masanori Okaniwa, Molly M. Stevens, Alfredo Alexander-Katz, Darrell J. Irvine

**Affiliations:** 1 Koch Institute for Integrative Cancer Research, Massachusetts Institute of Technology, Cambridge, MA USA; 2 Department of Materials, Imperial College London, London, UK; 3 Department of Bioengineering, Imperial College London, London, UK; 4 Institute of Biomedical Engineering, Imperial College London, London, UK; 5 Millennium Pharmaceuticals, Inc., Cambridge, MA USA; 6 Immunology Unit, Takeda Pharmaceutical Company Limited, Fujisawa, Kanagawa Japan; 7 Department of Biological Engineering, Massachusetts Institute of Technology, Cambridge, MA USA; 8 Department of Chemical Engineering, Massachusetts Institute of Technology, Cambridge, MA USA; 9 Oncology Drug Discovery Unit, Takeda Pharmaceuticals International Co., Cambridge, MA USA; 10 Department of Materials Science & Engineering, Massachusetts Institute of Technology, Cambridge, MA USA; 11 Ragon Institute of Massachusetts General Hospital, Massachusetts Institute of Technology and Harvard University, Cambridge, MA USA; 12 Howard Hughes Medical Institute, Chevy Chase, MD USA

**Keywords:** Immunotherapy, Self-assembly

## Abstract

Activation of the innate immune STimulator of INterferon Genes (STING) pathway potentiates antitumour immunity, but systemic delivery of STING agonists to tumours is challenging. We conjugated STING-activating cyclic dinucleotides (CDNs) to PEGylated lipids (CDN-PEG-lipids; PEG, polyethylene glycol) via a cleavable linker and incorporated them into lipid nanodiscs (LNDs), which are discoid nanoparticles formed by self-assembly. Compared to state-of-the-art liposomes, intravenously administered LNDs carrying CDN-PEG-lipid (LND-CDNs) exhibited more efficient penetration of tumours, exposing the majority of tumour cells to STING agonist. A single dose of LND-CDNs induced rejection of established tumours, coincident with immune memory against tumour rechallenge. Although CDNs were not directly tumoricidal, LND-CDN uptake by cancer cells correlated with robust T-cell activation by promoting CDN and tumour antigen co-localization in dendritic cells. LNDs thus appear promising as a vehicle for robust delivery of compounds throughout solid tumours, which can be exploited for enhanced immunotherapy.

## Abstract (web summary)

Here the authors investigate lipid nanodiscs as drug carriers for antitumour immunotherapy. They demonstrate that flexible lipid nanodiscs functionalized with STING-activating cyclic dinucleotides exhibit superior tumour penetration and tumour cell uptake compared with spherical liposomes, resulting in improved antitumour T-cell priming and tumour regression.

## Main

Immunotherapy treatments such as checkpoint blockade^1^ and chimeric antigen receptor T-cell therapies^2^ have revolutionized cancer patient care. However, intensive efforts are focused on expanding the repertoire of clinically viable immunostimulatory drugs to increase the response rate of immunotherapy^3^. One important class of agents is ligands for innate immune danger sensors, including Toll-like receptors, retinoic acid-inducible gene I (RIG-I)-like receptors, nucleotide-binding oligomerization domain (NOD)-like receptors and STimulator of INterferon Genes (STING)^4,5^. Delivery of such ‘danger signals’ to tumours has the potential to induce an in situ vaccination, where dying tumour cells are taken up by dendritic cells, which in turn are activated by the innate immune stimulators, leading to priming of de novo T-cell responses^6^. The danger sensor STING is particularly promising; preclinical studies have shown that STING activation, whether through the endogenous signalling pathway^7^ or by an exogenous agonist^8^, is key to promoting effective antitumour immunity^7^.

Therapeutic targeting of STING remains a substantial challenge. Intratumoral injection of the natural ligands for STING, cyclic dinucleotides (CDNs), has shown remarkable efficacy in preclinical studies and is under investigation in human clinical trials^5^. However, CDNs are hydrophilic small molecules, membrane impermeable and susceptible to rapid degradation by nucleases, making them unsuitable as agents for systemic administration^5^. Further, novel small-molecule STING agonist compounds in development^9–11^ are likely to face issues of toxicity without some means to concentrate their activity in the tumour microenvironment. This has led to the design of nanocarriers for STING agonist delivery based on liposomes, polymer particles or polymers that directly activate STING^12–16^. Liposomes and polymersomes have been administered systemically and shown to deliver CDNs to tumours^12–14,16^. However, these strategies led to CDN uptake in only a small fraction of cancer cells or tumour-infiltrating immune cells (∼2–10%)^14,16^, which may reflect their limited ability to diffuse through the dense tumour extracellular matrix (ECM) and propensity for clearance by the reticuloendothelial system. We hypothesized that a nanoparticle carrier better able to penetrate beyond the tumour vasculature would increase the likelihood of effective co-localization of CDNs with dying tumour cells following STING activation, ensuring optimal activation of dendritic cells for subsequent T-cell-mediated tumour immunity.

The size, shape, charge, surface chemistry and rigidity of nanoparticles are all parameters influencing tumour penetration^17,18^. Recent studies have emphasized the improved penetration capacity of nanomaterials with sizes <100 nm and high-aspect-ratio morphologies^19–23^. Based on their small size and deformable morphology, we predicted that lipid nanodiscs (LNDs), formed from the self-assembly of PEGylated lipids (PEG, polyethylene glycol) and high *T*_m_ (melting temperature) phospholipids^24,25^, could be particularly effective for tumour targeting.

Here we demonstrate that LNDs carrying CDNs are an effective immunotherapy following a single intravenous systemic administration in multiple tumour models. We compared treatment with CDN-conjugated PEGylated liposomes as a gold standard for approved nanomedicines. Through in vitro, in vivo and in silico experiments, we show that the unique properties of LND-CDN carriers enable superior tumour penetration and tumour cell uptake, resulting in improved antitumour T-cell priming and long-term tumour remission compared with high doses of free CDNs or CDN delivered by state-of-the-art liposomes.

## Results

### Design of PEG-LNDs for STING ligand delivery

**Fig. 1:**
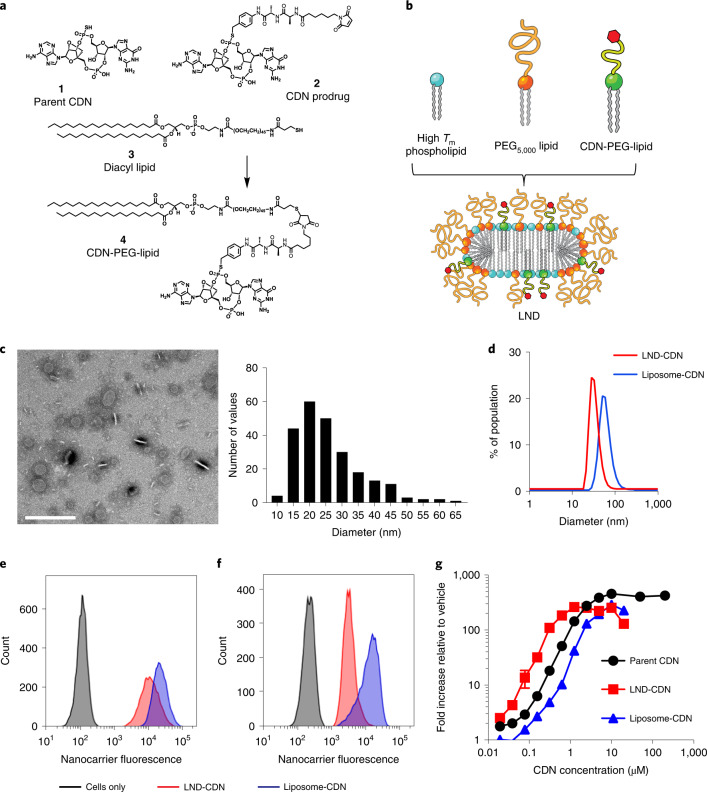
Design and characterization of nanoparticles for STING agonist delivery. **a**, Chemical structures of the parent CDN STING agonist (**1**), CDN prodrug (**2**), diacyl lipid (3) and CDN-PEG-lipid (**4**). **b**, Schematic of LND containing CDN-PEG-lipid. **c**, Negative stain transmission electron micrograph of LND-CDNs and histogram of measured LND diameters. Scale bar, 200 nm. This experiment was performed once. **d**, Dynamic light scattering analysis of LND-CDN (red) and liposome-CDN (blue) particle size distributions. **e**,**f**, Representative flow cytometry histograms showing uptake of fluorescent LND-CDN (red) or liposome-CDN (blue) by RAW-ISG cells (STING reporter cell line) (**e**) or MC38 tumour cells (**f**) following 24 h incubation at 37 °C with 5 µM CDN in each formulation. **g**, Dose–response curves showing STING activation in RAW-ISG reporter cells as measured by bioluminescence reporter relative to the vehicle-treated control following 24 h stimulation at 37 °C. Data are presented as mean values ± s.e.m. with *n* = 4 biologically independent samples for each concentration tested.

**Extended Data Fig. 1:**
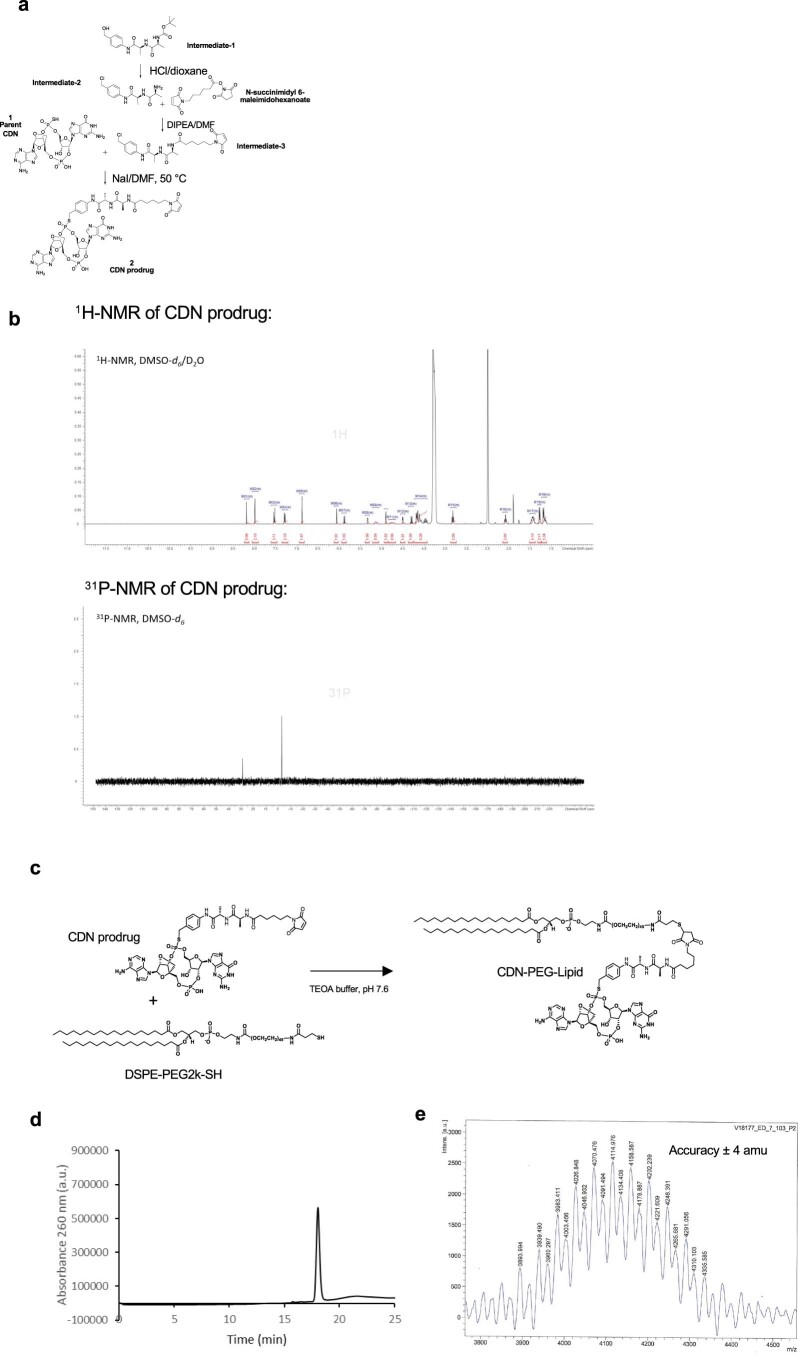
Characterization of CDN-PEG-lipid prodrug. **a**, Synthetic scheme describing the preparation of compound (2) (CDN prodrug) from parent CDN (1) and intermediate-3. **b**, ^1^H NMR and ^31^P-NMR spectra of CDN prodrug**. c**, Reaction scheme for production of CDN-PEG-lipid. **d**, HPLC analysis of CDN-PEG-lipid product. **e**, Mass spectrometry analysis of CDN-PEG-lipid.

**Extended Data Fig. 2:**
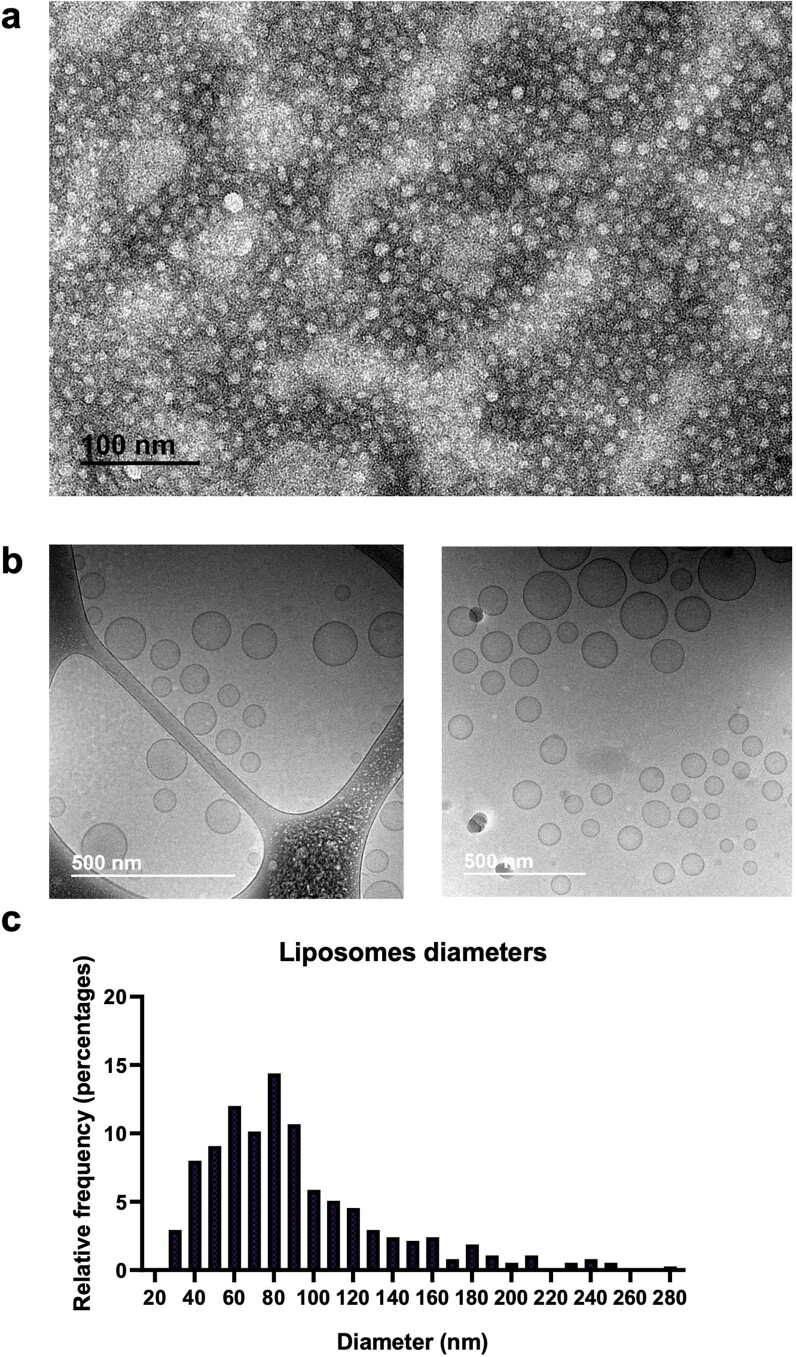
Characterization of CDN-liposomes. **a**, Representative TEM image of LND-CDN. **b**, Representative cryoTEM images of liposome-CDN. **c**, Size histograms of liposomes measured from cryoTEM. TEM results are representative of at least 2 independent experiments.

CDN prodrug **2** containing a dialanine peptide linker was synthesized and conjugated to a thiol-terminated PEG-phospholipid **3** via thiol–maleimide coupling (Fig. 1a and Extended Data Fig. 1a–e). The resulting CDN-PEG-lipid **4** was designed to facilitate formulation in lipid-based drug carriers, with release of the active STING agonist **1** upon peptidase cleavage in endosomes following cellular uptake^26^. We particularly focused on PEGylated LNDs (Fig. 1b), which form spontaneously when PEGylated lipids are combined at 20–30 mol% with high-*T*_m_ phospholipids in aqueous buffers^24^. LNDs were prepared via ethanol precipitation, leading to discoid morphologies with mean diameters of ∼26 nm and ∼33 nm as measured by transmission electron microscopy and dynamic light scattering, respectively (Fig. 1c,d, Supplementary Table 1 and Extended Data Fig. 2a). A subset of discs oriented perpendicular to the plane appear as rectangles with a width of 5–6 nm, as expected for a single lipid bilayer (Fig. 1c). For comparison, we prepared CDN-carrying vesicles with compositions similar to commercial long-circulating PEGylated liposomes. Liposomes as small as the LNDs prepared here are highly unstable^27^; we found that the smallest possible liposomal-CDN structures we could prepare were ∼60 nm diameter (Fig. 1d, Extended Data Fig. 2b,c and Supplementary Table 1). However, these vesicles serve as a benchmark representative of traditional nanomedicine formulations. Liposomes incorporating a few mol% of the CDN-PEG-lipid were stable, but showed a tendency to aggregate if the CDN was incorporated at 5 mol% or greater; we thus fixed the CDN-functionalized lipid at 5 mol% for LNDs and 1 mol% for liposome formulations.

**Extended Data Fig. 3:**
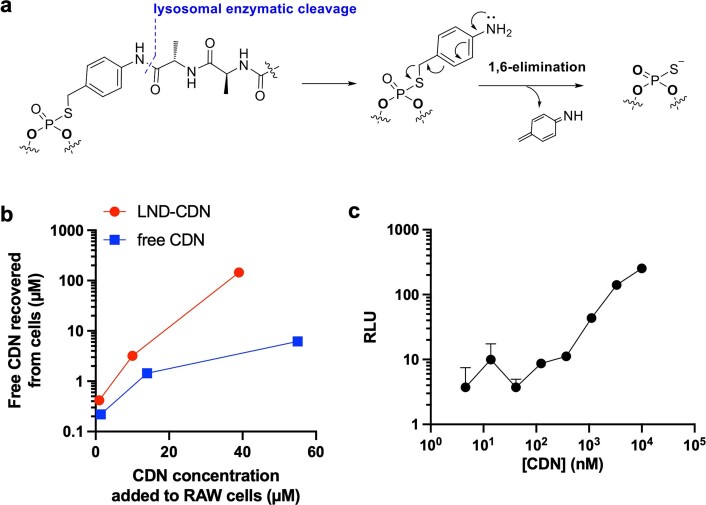
Release of CDN from the PEG-lipid prodrug in cells. **a**, Schematic of the chemistry of enzymatic cleavage of the dipeptide linker and subsequent self-immolative linker reaction. **b**, RAW macrophages were incubated for 18 hr with parent CDN or LND-CDN at varying concentrations. Cells were washed, then lysed and lysates were probed for quantity of parent CDN recovered by liquid chromatograph MS/MS analysis. **c**, Human THP-1-ISG reporter cells were incubated with indicated concentrations of LND-CDN for 3 hr, washed into fresh medium, then cultured for an additional 21 hr, followed by measurement of interferon-stimulated gene reporter activation by luciferase expression (n = 2 independent biological samples for each concentration and mean±s.e.m. is plotted; RLU, relative light units).

Fluorescently labelled LND-CDN or liposome-CDNs were readily taken up by mouse macrophages (RAW-ISG) and MC38 colon adenocarcinoma cells in cell culture (Fig. 1e,f). Guided by previous experience with antibody–drug conjugates, the CDN prodrug is designed to be cleaved following internalization into endolysosomes by cathepsins^28^, and subsequently undergo a rapid 1,6-elimination reaction to release the parent CDN (Extended Data Fig. 3a). To assess CDN release from LNDs, we incubated RAW cells with free CDN or LND-CDN for 18 h, then assessed release of the parent compound in the cells by liquid chromatography–tandem mass spectrometry. LND-CDN-treated cells showed substantial levels of free intracellular CDN, in fact at higher levels than cells incubated with parental CDN alone (Extended Data Fig. 3b). Using RAW-ISG reporter cells, we found that LND-CDN was a few-fold more potent than the liposome-CDN and the parent free CDN (Fig. 1g). However, at micromolar concentrations, all three forms of the drug fully activated the reporter cells. LND-CDN was also active in human THP-1 cells (Extended Data Fig. 3c).

### Modelling and in vitro tumour permeation of LNDs versus liposomes

**Fig. 2:**
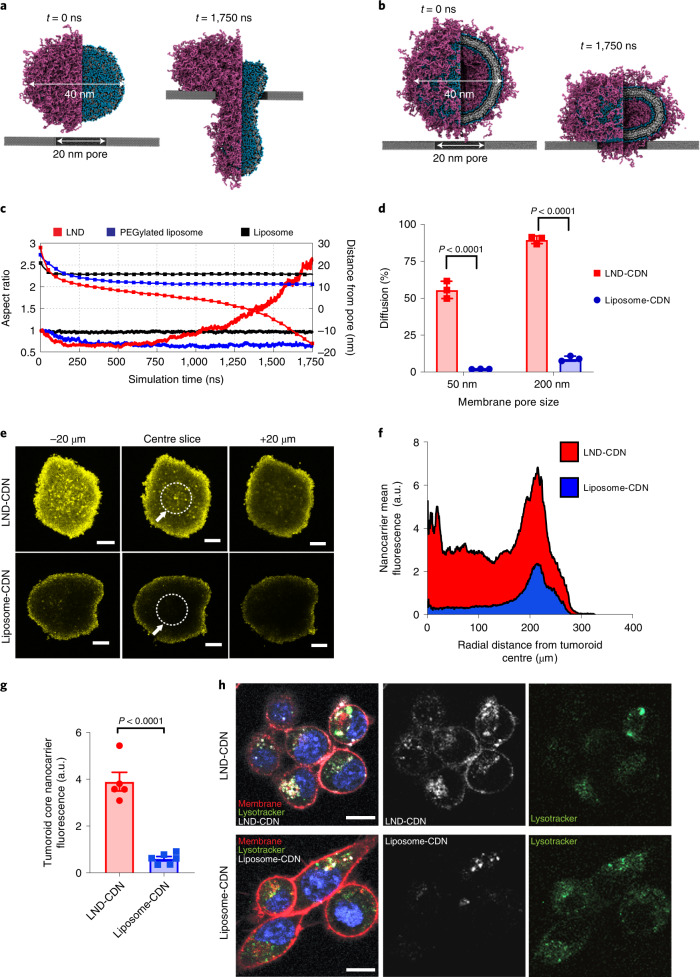
LND-CDN shows superior passive diffusion and tumour penetration compared with liposome-CDN in vitro. **a**,**b**, Coarse-grained simulation snapshots of an LND (**a**) and a PEGylated liposome (**b**), both with a diameter of 40 nm, before (*t* = 0 ns) and after (*t* = 1,750 ns) being pulled through a 20 nm pore by a force of 200 kJ mol^−1^ nm^−1^. Purple beads represent PEG polymers; blue, black and white beads correspond to lipid headgroups, glycerol groups and hydrophobic tails, respectively. **c**, Computed aspect ratio (lines) and distance of the particle centroid from the pore (squares) of LND, PEGylated liposome and bare liposome systems as a function of pulling simulation time. **d**, LND-CDN or liposome-CDN were added to a diffusion chamber at 0.5 µM (CDN concentration) separated from a receiver chamber by a 6 µm thick membrane with the indicated pore size and incubated at 25 °C. Shown is the percentage of particles detected in the receiver chamber after 24 h (mean ± s.e.m.). **e**–**g**, Fluorescent LND-CDN or liposome-CDN were added to the medium of wells containing MC38 tumour spheroids at 5 μM (CDN concentration) for 24 h, followed by washing to remove particles from the medium and imaging of particle penetration into spheroids by confocal microscopy (*n* = 5 independent spheroids for LND-CDN and *n* = 6 independent spheroids for liposome-CDN): representative spheroid *z*-stack images from the centre focal plane analysed and focal planes 20 µm above and below (**e**); radial distribution plots of nanocarrier fluorescence (**f**); and mean particle signal (±s.e.m.) measured in the central 100 µm radius core of spheroids (**g**, core region denoted by white dotted line and white arrow in **e**). **h**, MC38 cells were incubated for 4 h with 5 µM fluorescent liposome-CDNs or LND-CDNs, stained with membrane/nuclear (DAPI)/endosolysosomal (lysotracker) markers, and imaged by confocal microscopy. Shown are representative images from one of two independent experiments. Scale bars, 10 µm. Statistical comparisons in **d** and **g** performed using an unpaired, two-tailed Student’s *t*-test.

**Extended Data Fig. 4:**
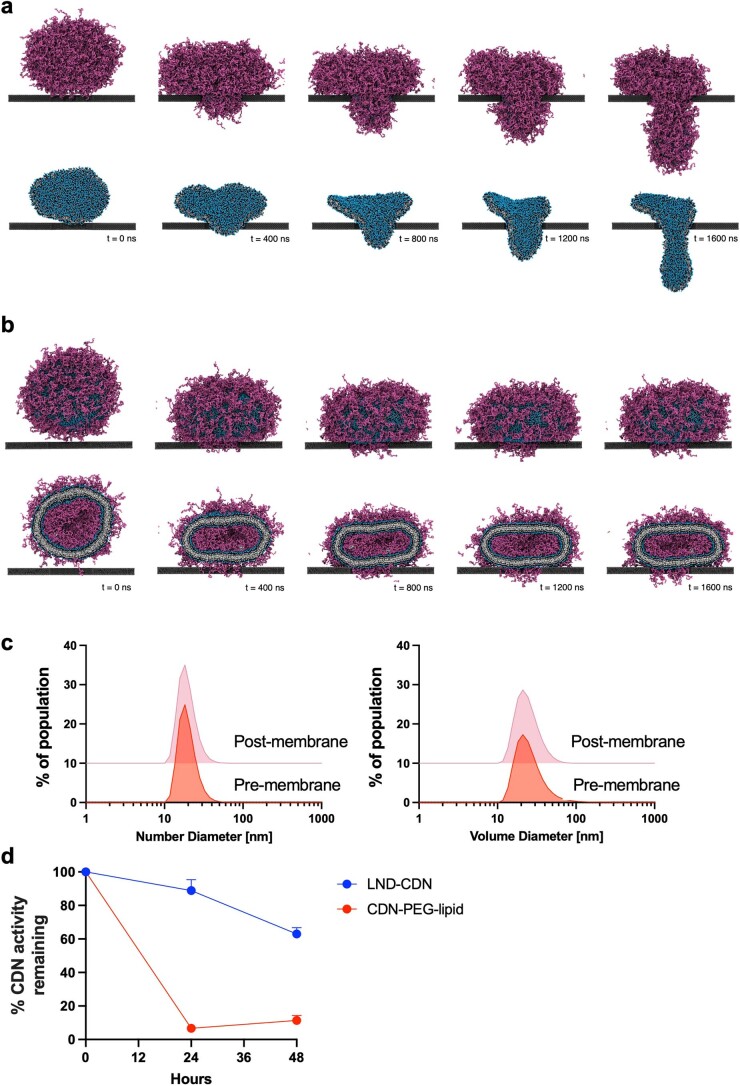
Stability and pore crossing by LND-CDN vs. liposomes. **a**, PEGylated LND with and without PEG molecules represented. **b**, PEGylated liposome without and with cross-sectional view of liposome interior. **c**, DLS analysis of LND size distributions before and after diffusion through 50 nm pore diameter membranes. **d**, LND-CDN or CDN-PEG-lipids were incubated with 10% serum in a dialysis cassette with a 5 KDa MWCO membrane, and STING activation bioactivity remaining in the sample (as assessed by activation of RAW-ISG reporter cells) was measured over time (n = 3 biologically independent samples per timepoint, mean±s.e.m. is plottted).

To gain insight into the potential differences in transport behaviour of LNDs versus liposomes, we performed coarse-grained molecular dynamics simulations on a model LND (diameter, 40 nm) being pulled through a small rigid pore 20 nm in diameter, compared to a model PEGylated or ‘bare’ liposome of similar lipid composition and of the same diameter (Fig. 2a,b). Even under a modest pulling force of ∼330 pN (200 kJ mol^−1^ nm^−1^), the LND was able to deform and enter a pore smaller than its equilibrium diameter, whereas the liposome was unable to deform sufficiently to enter the pore (Fig. 2a-c, Extended Data Fig. 4a,b and Supplementary Videos 1–3). To establish the important differences between LNDs and liposomes we also developed an analytical model that captures the essential elements of LND translocation through pores (Supplementary Modelling Discussion). These calculations suggest that in many cases where the size of the disc is slightly larger than the size of the constriction (for example, between 10% and 20%), the translocation forces approach thermal forces which are approximated to be of the order of ∼0.1 pN for a 30 nm LND, implying such discs might diffuse much more rapidly than harder objects of the same size, even without the need for external forces/flows to drag them through.

To assess experimentally the comparative ability of LNDs versus liposomes to pass through pores of defined sizes, we considered nanoparticle transport across track-etched membranes. LND-CDNs efficiently crossed membranes with well-defined pore sizes of 50 or 200 nm, and retained their same size distribution before/after membrane transit (Fig. 2d and Extended Data Fig. 4c). By contrast, although liposome-CDNs readily passed through 200 nm syringe filters under gentle pressure, they were inefficient at translocating through either pore size by passive diffusion. We next compared LND versus liposome penetration of MC38 tumour spheroids in vitro. Fluorophore-labelled LND-CDNs or liposome-CDNs were added to the culture medium of spheroids, and tumour penetration was tracked by three-dimensional (3D) confocal microscopy (Fig. 2e,f). Tumouroids treated with LNDs displayed significantly higher signals in their core compared with liposomes (Fig. 2e-g). Motivated by these observations, we also examined uptake of liposome-CDNs and LND-CDNs with isolated MC38 cells. While liposomes showed predominant co-localization with endolysosomal compartments, LND-CDNs showed both uptake into endosomal compartments and association with the cell membranes, suggesting that nanodiscs may be capable of binding to or fusing with the plasma membrane prior to endocytosis (Fig. 2h). To assess the stability of LND-CDN in the presence of serum proteins, we incubated free CDN-PEG-lipid or LND-CDNs with 10% serum and measured their STING activation bioactivity over time. While bioactivity of free CDN-PEG-lipid fell to near baseline within 24 h, LND-CDNs retained 75% bioactivity over 48 h, suggesting significant stability in the presence of serum (Extended Data Fig. 4d). Altogether, these findings suggested nanodiscs might have significant advantages relative to traditional liposomes in delivering STING agonists deeply throughout a tumour bed.

### Biodistribution of LND-CDNs versus liposome-CDNs

**Fig. 3:**
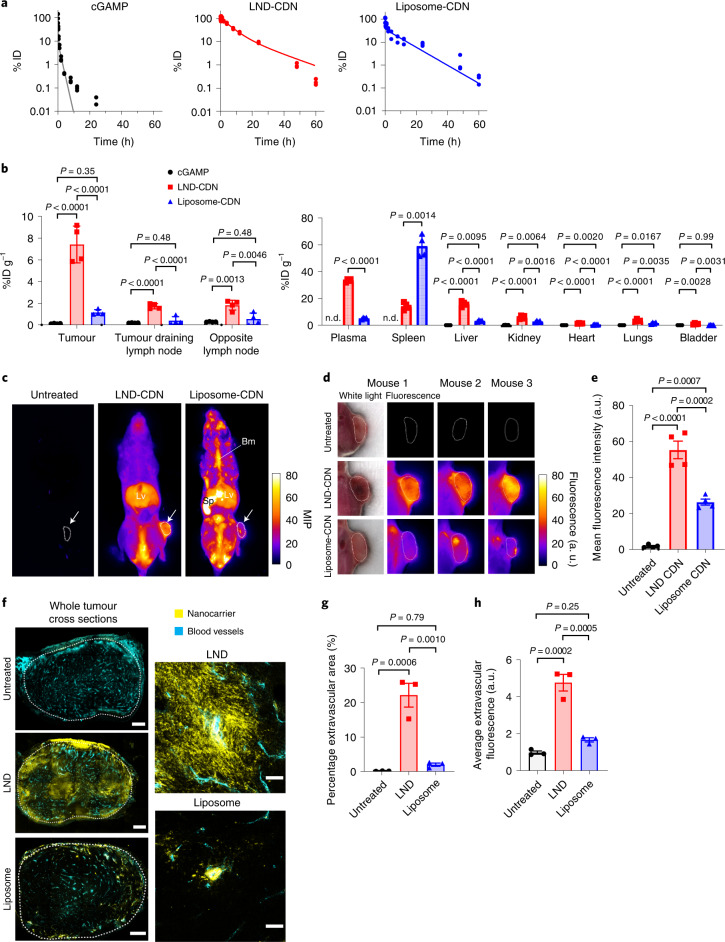
LND-CDN exhibits efficient tumour penetration *in vivo*. **a**, C57Bl/6 mice (*n* = 3 animals per group) were injected intravenously with Cy5-labelled cGAMP, Cy5-labelled LND-CDN or liposome-CDN (all at 5 nmol CDN) and plasma levels were quantified by fluorescence measurements over time. Dotted lines show two-phase decay curve fits. **b**, C57Bl/6 mice (*n* = 4 animals per group) were inoculated in the flank with 5 × 10^5^ MC38 tumour cells, and 10 days later, 5 nmol Cy5-labelled cGAMP, LND-CDN or liposome-CDN were administered intravenously. Shown is the organ-level biodistribution (mean ± s.e.m.) determined from fluorescence measurements on digested tissues 24 h later. n.d., not detectable. **c**–**e**, MC38-tumour-bearing mice (*n* = 4 animals per group) were treated as in **b** with 5 nmol near-infrared dye-labelled LND-CDN, liposome-CDN or left untreated, and then sacrificed at 4 h. The mice were rapidly frozen and then imaged by cryofluorescence tomography with 50 µm serial sections. **c**, Representative maximum intensity projections (MIP) of whole mice with tumours identified with a white arrow and outlined with a dotted white line. Lv, liver, Sp, spleen; Bm, bone marrow. **d**, Enlarged images of a single slice from the middle of representative tumours with the corresponding white-light image shown only for mouse 1. **e**, Mean fluorescence intensities (±s.e.m.) averaged from three tumour regions of interest per mouse (one at the tumour centre, one 1 mm dorsal and one 1 mm ventral) (*n* = 4 mice per group). **f**, MC38-tumour-bearing mice (*n* = 3 animals per group) were treated with LND or liposomes as in **b** and tumours were excised 24 h later for histology. High-molecular-weight fluorescein isothiocyanate–dextran (cyan) was injected intravenously 10 min before the mice were sacrificed to label vasculature. Shown are representative whole tumour cross sections and enlarged views of tumour vessels from mice treated with Cy5-labelled LND or liposome (yellow). Scale bars: whole tumour cross sections, 500 µm; enlarged view, 50 µm. **g**,**h**, The percentage of the extravascular tumour area with nanoparticle fluorescence (**g**) and the average fluorescence intensity of the extravascular tumour area (**h**) was quantified (mean ± s.e.m.). Each point represents one mouse and is the average of two unique tumour cross sections. Statistical comparisons in **b**, **e**, **g**, and **h** were tested using an ordinary one-way analysis of variance (ANOVA) with Tukey’s multiple-comparisons test.

We measured the pharmacokinetic profiles of Cy5-conjugated cGAMP (a labelled surrogate of the parental CDN), fluorophore-labelled LND-CDNs and liposome-CDNs following intravenous administration. cGAMP-Cy5 cleared rapidly with a terminal half-life of ∼1 h, whereas LNDs and liposomes displayed extended circulation half-lives of 12.6 and 7.6 h, respectively (Fig. 3a). Biodistribution analysis at 24 h in animals bearing established MC38 tumours revealed substantially higher tumour accumulation of LND-CDNs (7.4% ID g^−1^) compared to liposome-CDNs (1.1% ID g^−1^, Fig. 3b). LNDs also accumulated in tumour-draining and non-tumour-draining inguinal lymph nodes, but exhibited low uptake in other tissues; free cGAMP was either not detected or below 0.5% ID g^-1^ in any tissue. As expected, the spleen and liver were sites of uptake for both lipid formulations; liposome-CDNs accumulated to high levels in the spleen while LND-CDNs showed greater uptake in the liver.

We next performed whole-animal cryofluorescence tomography (CFT) to image the whole-animal biodistribution of LND- or liposome-CDNs 4 h post-administration. LNDs accumulated in the liver and throughout tumour cross sections (Fig. 3c–e and Supplementary Video 4). By contrast, liposome-CDNs concentrated in the spleen and bone marrow with only patchy accumulation in tumours; total tumour accumulation was notably lower than LNDs (Fig. 3c–e and Supplementary Video 5). Traditional histology 24 h post-injection corroborated the observations made by CFT, showing dispersal of LNDs throughout tumours while liposomes exhibited minimal dispersal from blood vessels (Fig. 3f–h). Thus, LND-CDNs exhibited both greater total accumulation in tumours and greater penetration through the tumour bed than PEGylated liposome-CDNs.

### Therapeutic efficacy and safety of LND-CDNs

**Fig. 4:**
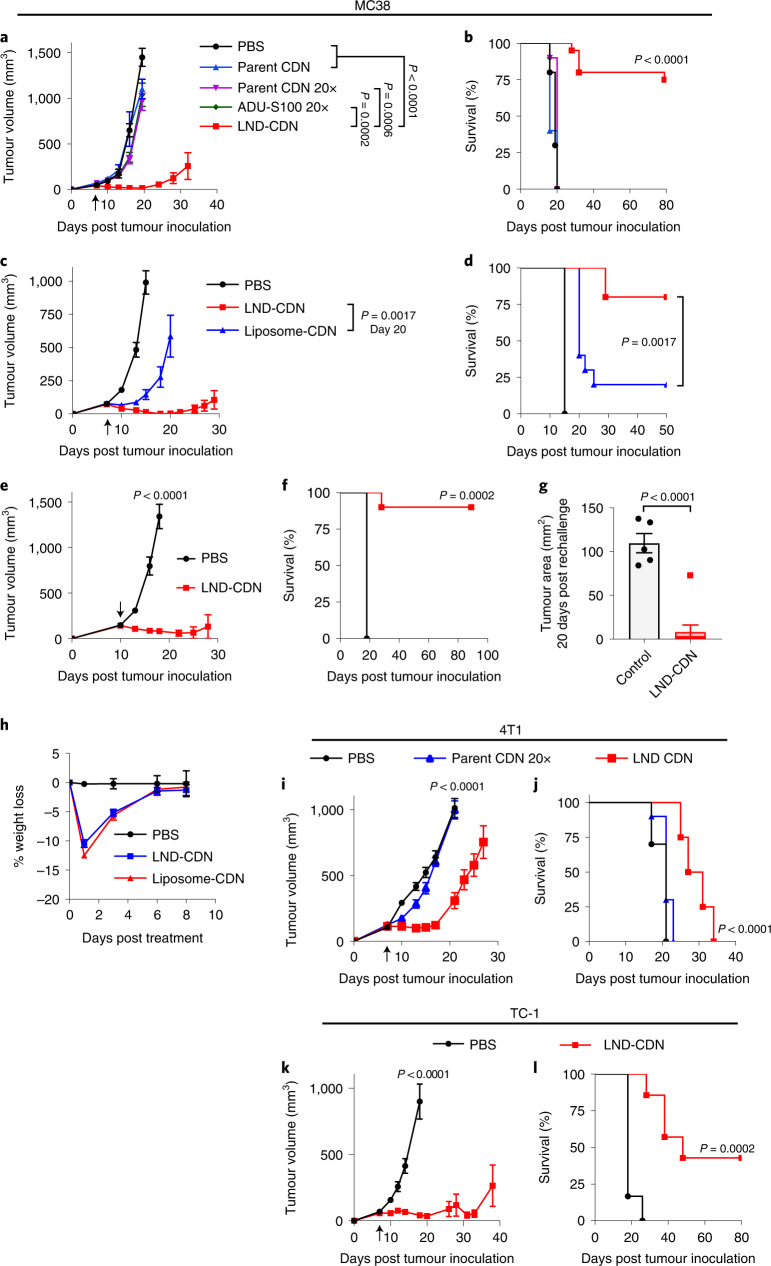
A single dose of LND-CDN shows therapeutic efficacy in multiple syngeneic tumour models. **a**–**d**, C57Bl/6 mice were inoculated with 5 × 10^5^ MC38 tumour cells and then treated on day 7 with intravenous administration of PBS vehicle (*n* = 10), parent CDN (5 nmol per mouse, *n* = 5), parent CDN (100 nmol per mouse, *n* = 10), ADU-S100 (100 nmol per mouse, *n* = 10) or LND-CDN (5 nmol per mouse, *n* = 20): tumour size (**a**, mean ± s.e.m) and overall survival (**b**); or PBS vehicle (*n* = 9), LND-CDN (5 nmol per mouse, *n* = 10) or liposome-CDN (5 nmol per mouse, *n* = 10): tumour size (**c**, mean ± s.e.m.) and overall survival (**d**). **e**,**f**, Mice with MC38 tumours as in **a** were treated on day 10 with PBS vehicle (*n* = 5) or LND-CDN (5 nmol per mouse, *n* = 10): tumour growth (**e**) and survival (**f**). **g**, Mice (*n* = 9 animals per group) that rejected their tumour following treatment in **e**,**f** were rechallenged with 5 × 10^5^ MC38 tumour cells 90 d following the initial tumour inoculation on the opposite flank and tumour growth was assessed 20 d later (mean ± s.e.m.), compared to naive age-matched control mice (*n* = 5) given the same tumour challenge. **h**, C57Bl/6 mice bearing MC38 tumours (*n* = 5 animals per group) were treated as in **c** and animal weights were tracked over time. **i**,**j**, Tumour growth (**i**, mean ± s.e.m.) and survival (**j**) curves of BALB/c mice (*n* = 10 animals per PBS and parent CDN groups, *n* = 8 animals per LND-CDN group) implanted orthotopically in the mammary fat pat with 5 × 10^5^ 4T1-Luc tumour cells and then treated intravenously on day 7 with PBS vehicle, parent CDN (200 nmol) or LND-CDN (10 nmol). **k**,**l**, C57Bl/6 mice were inoculated in the flank with 3 × 10^5^ TC-1 tumour cells and treated intravenously on day 7 with PBS vehicle (*n* = 6) or LND-CDN (5 nmol, *n* = 7): shown are tumour growth (**k**, mean ± s.e.m.) and survival (**l**). Statistical comparisons among tumour sizes in **a**, **c**, **e**, **i** and **k** were tested using an ordinary one-way ANOVA with Tukey’s multiple-comparisons test and in **g** using an unpaired, two-tailed Student’s *t*-test. Statistical comparisons between survival curves were performed using a log-rank (Mantel–Cox) test.

In preliminary therapeutic studies, we established 5 nmol (dose of CDN) as a maximum tolerated dose for the LND-CDNs in the setting of single-dose treatment, above which animals showed prolonged lethargy and weight loss. Using this dose, mice bearing established MC38 flank tumours were treated with a single dose of LND-CDNs, the equivalent dose of free parental CDNs, a 20-fold higher dose of parent CDNs (100 nmol) or 100 nmol of ADU-S100, a STING agonist currently in clinical trials. As shown in Fig. 4a,b, the LND-CDNs were strikingly effective, eliciting tumour regression and complete responses in 75% of the animals, while the free CDN compounds at either dose were completely ineffective. Liposome-CDNs triggered tumour growth delay lasting a few days, but the tumours rebounded and grew out in a majority of animals starting around one week after dosing with liposomes, while LND-CDNs triggered steady regression and rejection of a majority of tumours (Fig. 4c,d). Delaying treatment until day 10, when MC38 tumours had a mean size of ∼140 mm^3^, LND-CDN therapy was still effective, with a majority of animals exhibiting complete tumour rejection (Fig. 4e,f). Eight of nine cured LND-treated mice rejected a rechallenge with MC38 cells on the opposite flank 90 d after the original tumour inoculation, demonstrating the formation of antitumour immune memory (Fig. 4g).

**Extended Data Fig. 5:**
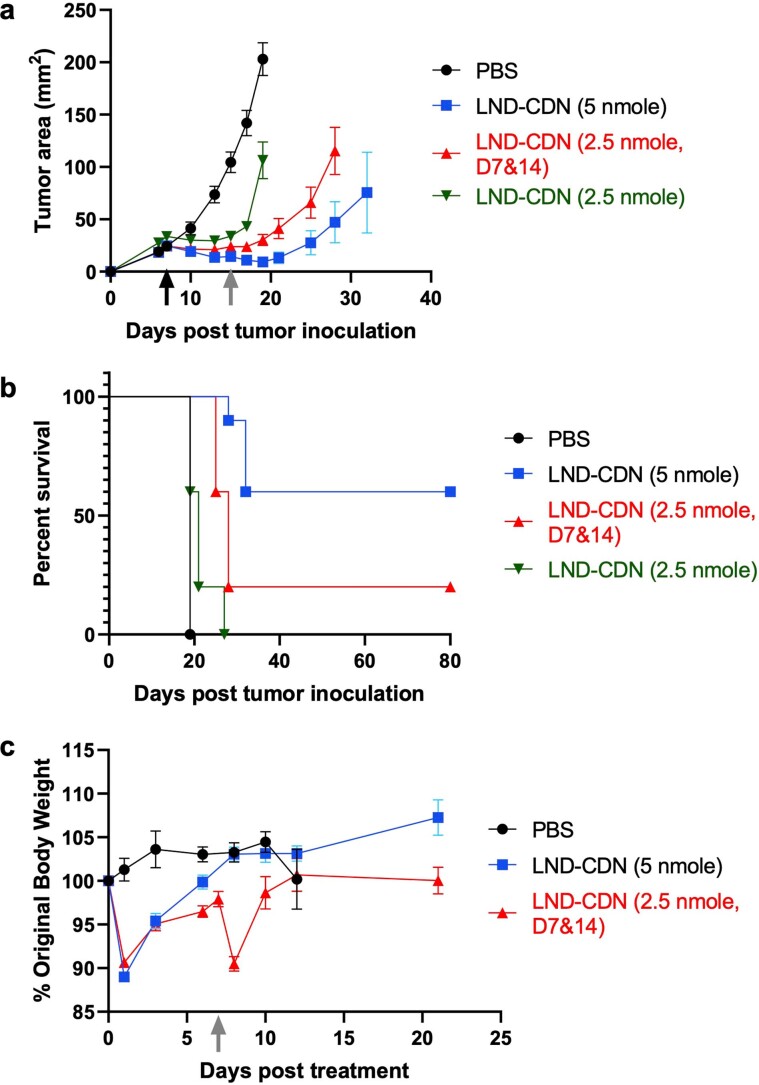
Lower doses of LND-CDN exhibited similar transient toxicity but substantially reduced efficacy. Groups of C57Bl/6 mice (*n* = 10 animals/group) were inoculated with 3 × 10^5^ MC38 tumour cells s.c. in the flank on day 0, then treated on day 7 with a single dose of 5 nmol LND-CDN, a single dose of 2.5 nmol LND-CDN, two doses at days 7 and 14 of 2.5 nmol LND-CDN, or saline control. Shown are tumour growth curves (mean ± s.e.m.) (**a**), overall survival (**b**), and weight loss (mean ± s.e.m.) (**c**) of animals over time.

**Extended Data Fig. 6:**
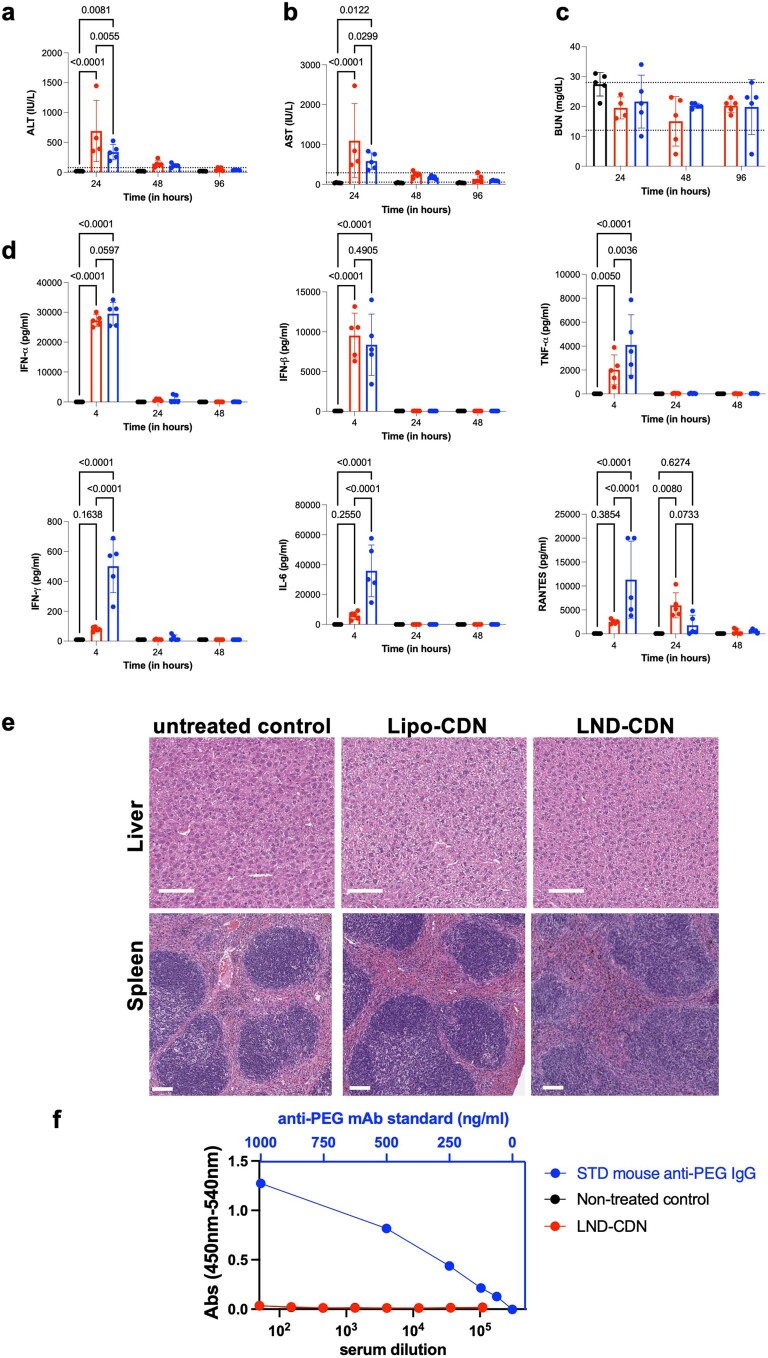
Systemic responses to LND-CDN and liposome-CDN administration. **a-e**, Groups of C57Bl/6 mice (*n* = 5 animals/group) were treated once by i.v. injection with 5 nmol LND-CDN (red), 5 nmol liposome-CDN (blue), or saline control (black). Shown are serum concentrations of liver enzymes (**a**) alanine aminotransferase, (**b**) aspartate aminotransferase, and (**c**) blood urea nitrogen with the normal ranges indicated by dashed horizontal lines. **d**, Inflammatory cytokines and chemokines measured by cytokine bead array as a function of time. **e**, Liver and spleens were collected at 48 hr post dosing for histopathological imaging. Scale bars 100 µm. **f**, Groups of C57Bl/6 mice (*n* = 6 (Non-treated control) or 8 (LND-CDN) animals/group) were inoculated with 3 × 10^5^ MC38 tumour cells s.c. in the flank on day 0, then treated on days 7, 14, and 21 with 5 nmol LND-CDN. Serum was collected on day 28 for ELISA analysis of anti-PEG IgG. Shown are raw ELISA absorbances as a function of serum dilution (bottom x-axis) and binding of serial dilutions of a monoclonal anti-PEG antibody standard (‘STD mouse anti-PEG IgG’. top x-axis). Data are shown as mean ± SEM and analysed by two-way ANOVA with Tukey post-test statistical analysis: ns, not significant; *, p < 0.05; **, p < 0.01; ****, p < 0.0001.

In response to treatment with either LNDs or liposomes, mice displayed a transient weight loss that recovered within a few days (Fig. 4h). Nanodiscs administered at lower doses or administered as two smaller doses of 2.5 nmol each showed similar transient toxicity but substantially reduced efficacy (Extended Data Fig. 5a–c). Serum ALT/AST levels and inflammatory cytokines/chemokines were transiently elevated after dosing, but returned to baseline within 24–48 h (Extended Data Fig. 6a–d). As spleen and liver were the primary sites of LND-CDN and liposome-CDN accumulation outside of tumours, we carried out histopathological analysis of these organs 48 h after dosing in tumour-bearing mice. As shown in Extended Data Fig. 6e, liver and spleen tissues of treated mice were indistinguishable from untreated animals.

We next evaluated LND-CDN treatment in additional tumour models. In the aggressive orthotopic 4T1 breast cancer model, LND therapy delayed tumour outgrowth and increased median survival compared with untreated tumours, while the parent CDNs at a 20-fold higher dose had no effect (Fig. 4i-j). In the TC-1 model of human papilloma virus-driven cancers, LND therapy led to tumour regressions and cures of ∼43% of mice (Fig. 4k–l). Hence, LND delivery of CDNs appears to be safe and efficacious in multiple syngeneic tumour models. While we focused here on examining responses to single-dose treatment given its unexpected high level of efficacy, a concern in clinical translation would be the potential for the heavily PEGylated LND-CDNs to promote an anti-PEG antibody response following repeated dosing that could lead to allergic reactions in patients and/or decreased efficacy^29,30^. Thus, we carried out a repeat dosing study in the MC38 model, dosing LND-CDNs three times at weekly intervals, and measuring anti-PEG IgG by enzyme-linked immunosorbent assay (ELISA) one week after the final dose. This study revealed no evidence for a humoral response against PEG, suggesting a lack of immunogenicity of the carrier in this treatment setting (Extended Data Fig. 6f).

### Acute responses to LND-CDN or liposome-CDN therapy

**Fig. 5:**
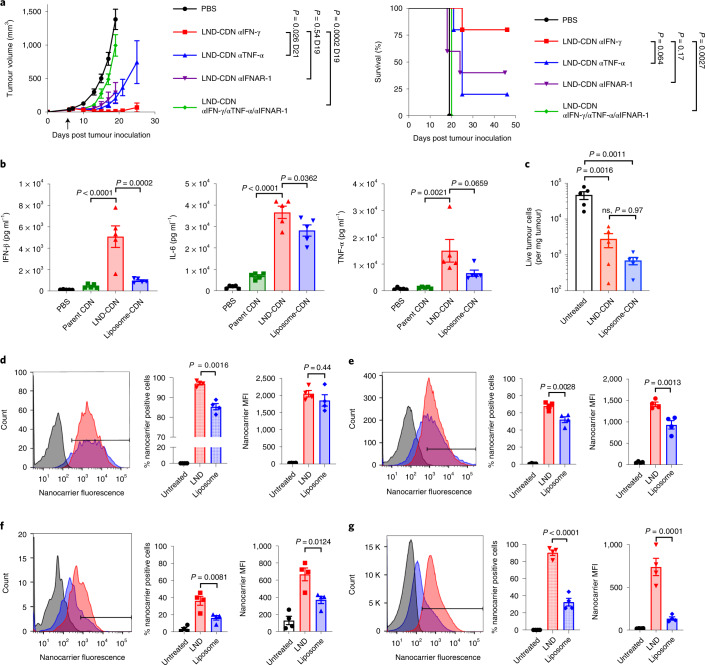
LND-CDN enhances cytokine production in tumours and delivery of CDN to tumour cells. **a**, Tumour growth (mean ± s.e.m.) (left) and survival for mice (*n* = 5 animals per group) (right) bearing MC38 flank tumours treated with LND-CDN as in Fig. 4a in the presence of neutralizing antibodies against IFN-γ (αIFN-γ), TNF-α (αTNF-α) or IFNAR-1 (αIFNAR-1). **b**, Mice (*n* = 5 animals per group) bearing MC38 tumours as in **a** were treated with 5 nmol parent CDN, LND-CDN or liposome-CDN and cytokine levels (mean ± s.e.m.) in tumour lysates were assessed 4 h later by bead-based ELISA. **c**, The number of live tumour cells per mg of tumour (mean ± s.e.m.) was quantified by flow cytometry 24 h after treatment with LND-CDN or liposome-CDN, compared with untreated tumours (*n* = 5 mice per group). **d**–**g**, MC38-tumour-bearing mice (*n* = 4 animals per group, mean ± s.e.m. values are shown in bar graphs) as in **a** were administered Cy5-labelled LND or PEGylated liposomes, and uptake in cells isolated from tumours was assessed 24 h later by flow cytometry: shown are representative histograms, percentage nanoparticle-positive cells and mean fluorescence intensity for tumour endothelial cells (**d**), CD11b^+^CD11c^-^ myeloid cells (**e**), CD11c^+^CD11b^−^ dendritic cells (**f**) and CD45^−^ non-endothelial cells (**g**). Statistical comparisons among tumour areas in **a** and in **b**–**g** were performed using one-way ANOVA with Tukey’s multiple-comparisons test and survival curves in **a** were compared using a log-rank (Mantel–Cox) test.

**Extended Data Fig. 7:**
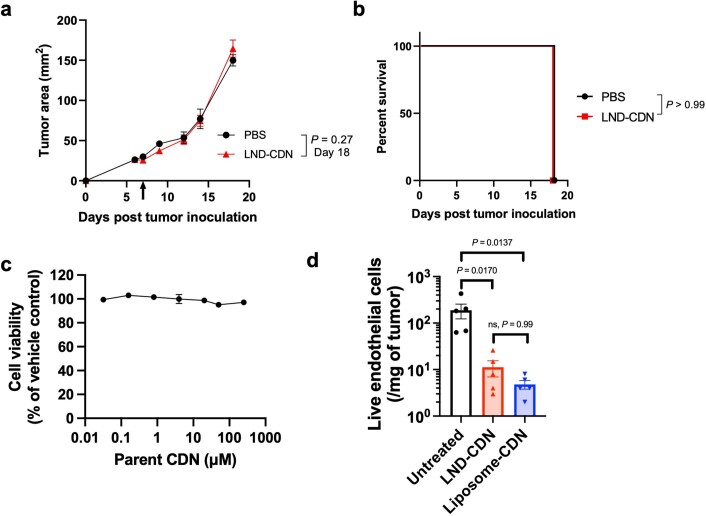
LND-CDN treatment of *STING*^-/-^ mice and CDN response of MC38 cells *in vitro*. **a**,**b**, Groups of *STING*-/- mice on the C57Bl/6 background (*n* = 6 (PBS) or 5 (LND-CDN) animals/group) were inoculated with 5 × 10^5^ MC38 tumour cells in the flank. Seven days later, animals were treated once by i.v. injection with 5 nmol LND-CDN or saline control. Shown are tumour growth (mean ± s.e.m.) (**a**) and overall survival (**b**). Statistical comparisons between tumour growth curves were made by an unpaired, two-tailed Student’s t-test; survival curves were statistically compared using a log-rank (Mantel–Cox) test. (**c**) MC38 tumour cells were incubated with a range of parent CDN concentrations in complete media for 24 hours and, subsequently, cell viability was assessed using a resazurin-based *in vitro* toxicology assay kit (Millipore Sigma) as per the manufacturer’s instructions. Each point represents the mean of four replicates (±s.e.m.). **d**, The number of live tumour endothelial cells per milligram of tumour (mean ± s.e.m.) was quantified by flow cytometry 24 h after treatment of MC38 tumours with LND-CDN or liposome-CDN, compared to untreated tumours (n = 5 mice per group). Statistical comparisons were made with a one-way ANOVA with Tukey’s multiple comparisons test.

In agreement with previous studies showing that antitumour effects of STING agonists are dependent on STING expression in host cells^8^, LND-CDN treatment of STING^−/−^ mice bearing MC38 tumours was ineffective (Extended Data Fig. 7a,b). Interferon β (IFN-β) and tumour necrosis factor α (TNF-α) are also important factors for CDN therapy^31,32^. LND-CDN therapy in the presence of blocking antibodies against the type I interferon receptor (IFNAR-1) or TNF-α, but not IFN-γ, led to substantial reductions in LND-CDN efficacy; blocking all three of these factors simultaneously led to complete treatment failure (Fig. 5a). We next compared the ability of LND- versus liposome-CDNs to activate early cytokine production in tumours. As shown in Fig. 5b, 4 h after administration of LND-CDN or liposome-CDN to MC38-tumour-bearing mice, high levels of interleukin 6 (IL-6) and TNF-α were measured in tumours by either formulation, but LND-CDNs triggered much higher levels of IFN-β production (Fig. 5b).

**Extended Data Fig. 8:**
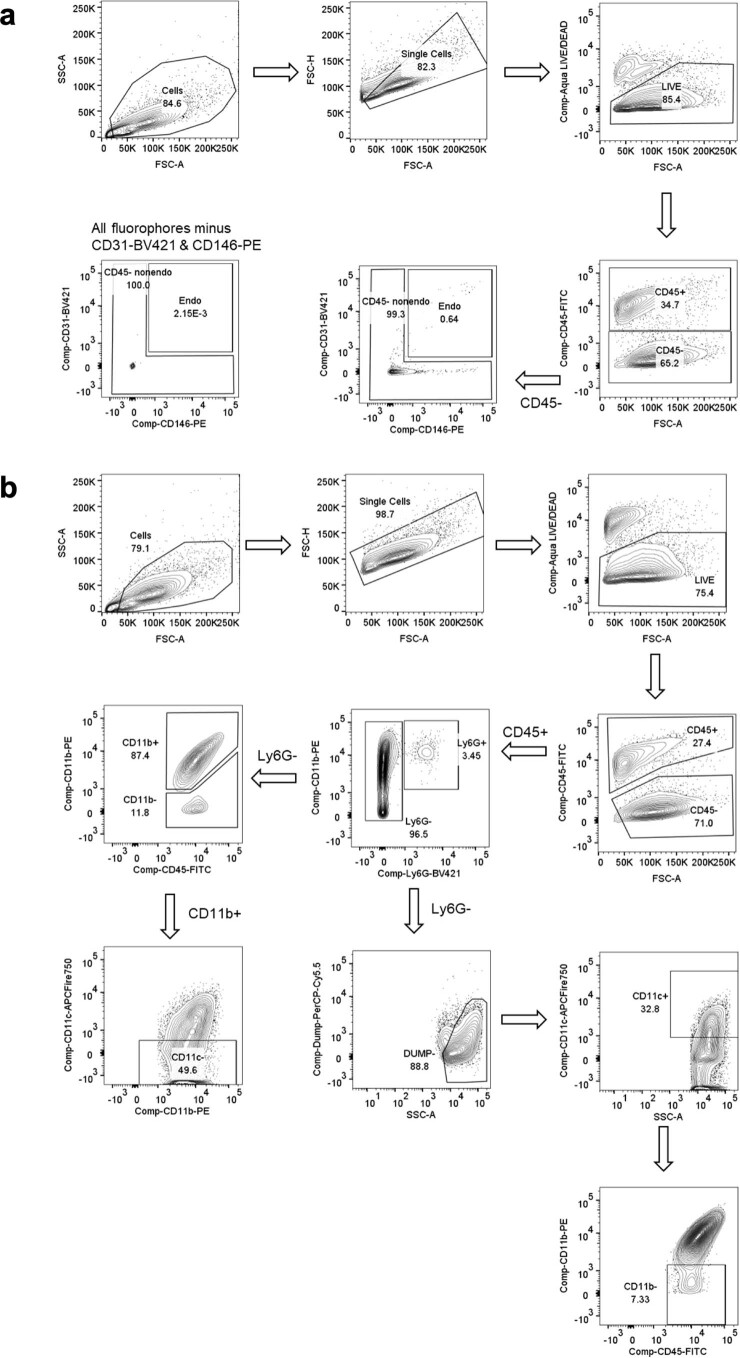
Flow cytometry gating strategy for identifying tumour endothelial cells, tumour cells, and myeloid cells. **a**, Flow cytometry gating strategy to identify tumour endothelial cells (CD45^−^CD31^+^ CD146^+^) and non-endothelial tumour cells (all other CD45^−^ cells) is show (see methods for details on tumour digestion and the antibodies used for staining). **b**, Gating strategy to identify tumour myeloid subsets referred to as CD11b^+^CD11c^−^ cells (CD45^+^ Ly6G^−^ CD11b^+^ CD11c^−^) and CD11c^+^ CD11b^−^cells [CD45^+^ Ly6G^−^DUMP(CD19 CD3e NK1.1)^-^ CD11c^+^ CD11b^−^] is show (see methods for details on tumour digestion and the antibodies used for staining).

STING activation can be cytotoxic in some cancer cells, but did not induce direct MC38 cell death in vitro (Extended Data Fig. 7c). STING activation can trigger rapid death of tumour endothelial cells, leading to profound early tumour necrosis^31,32^. We found that both LND-CDN and liposome-CDN treatment triggered massive cell death in tumour cells and tumour endothelial cells 24 h post-administration, suggesting that both formulations were effective at eliciting this first step of STING activity (Fig. 5c and Extended Data Fig. 7d). We next treated MC38 tumours with fluorophore-labelled liposomes or LNDs in the absence of the STING agonist cargo (to avoid confounding effects of cell death), and analysed tumours 24 h later by flow cytometry (Extended Data Fig. 8a,b). Both LNDs and liposomes were taken up by the majority of tumour endothelial cells and tumour myeloid cells (Fig. 5d,e). However, LNDs accumulated ∼2-fold more in CD11c^+^ dendritic cells (DCs) and uptake in CD45^−^, non-endothelial cells (the vast majority of which are the cancer cells) was notably greater for the LNDs (Fig. 5f,g). Thus, while both LND and liposomes reached tumour endothelial cells, only LNDs effectively reached the majority of cancer cells.

### Role of innate and adaptive immune cells in LND-CDN therapy

**Fig. 6:**
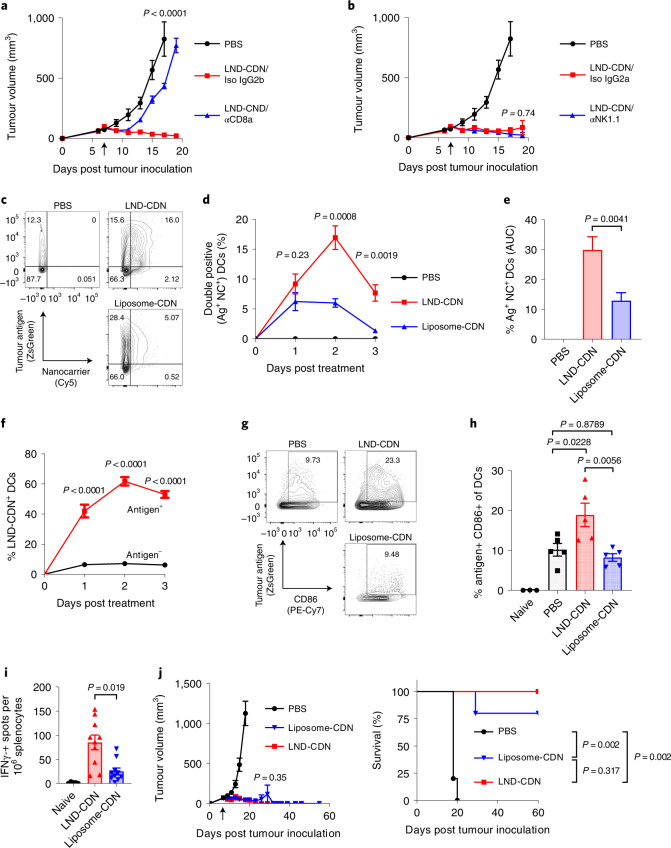
Co-localization of tumour antigen and LND-CDN nanoparticles in lymph node dendritic cells leads to effective antitumour T-cell priming. **a**,**b**, Mice with MC38 tumours (*n* = 5 animals per group) were treated as in Fig. 4a. Depleting antibodies against CD8 (αCD8) (**a**) or NK1.1 (αNK1.1) (**b**), or their respective isotype control antibodies (Iso), were administered on days 6, 8, 11 and 15 after tumour inoculation. The graphs show the average tumour growth versus time (error bars, s.e.m.) and the common PBS control group is shown in both graphs for clarity. **c**–**h**, C57Bl/6 mice (*n* = 5 animals per group) were inoculated with 5 × 10^5^ MC38-ZsGreen tumour cells in the flank, and 7 d later were left untreated or treated with Cy5-labelled LND-CDN or liposome-CDN (5 nmol CDN). Between 1 to 3 d later, TDLNs were isolated for flow cytometry analysis. Shown are representative flow cytometry plots of tumour antigen and nanoparticle uptake in DCs at day 2 (**c**), mean ± s.e.m. percentages of tumour antigen ZsGreen^+^NP^+^ DCs (**d**), area-under-the-curve (AUC) of Ag^+^NP^+^ DCs over time (**e**), analysis of mean ± s.e.m. percentages of LND-CDN^+^ DCs that are Ag^+^ or Ag^-^ (**f**), representative flow cytometry plots of tumour antigen uptake and CD86 upregulation in DCs at day 3 (**g**), and mean ± s.e.m. percentages of tumour antigen ZsGreen^+^CD86^+^ DCs (**h**). **i**, MC38-tumour-bearing mice (*n* = 10 animals per group) were treated with LND-CDN or liposome-CDN as in **a**, and tumour-specific T cells were assayed by IFN-γ ELISPOT 14 d following treatment. **j**, MC38-tumour-bearing mice (*n* = 5 animals per group) were treated on day 7 by intratumoral injection of 5 nmol LND-CDN or liposome-CDN. Shown are mean ± s.e.m. tumour area and survival. Statistical analysis of tumour growth in **a**, **b** and **j** was performed using one-way ANOVA with Tukey’s multiple-comparisons test. Statistical comparisons among cell percentages and AUCs in **d**–**f** and **h**, and tumour growth in **j** (day 18) were tested using an ordinary one-way ANOVA with Tukey’s multiple-comparisons test. Statistical comparisons among groups in **i** were tested using Brown–Forsythe and Welch’s ANOVA tests with Dunnett’s T3 multiple-comparisons test. Statistical comparisons between survival curves in **j** were performed using a log-rank (Mantel–Cox) test.

**Extended Data Fig. 9:**
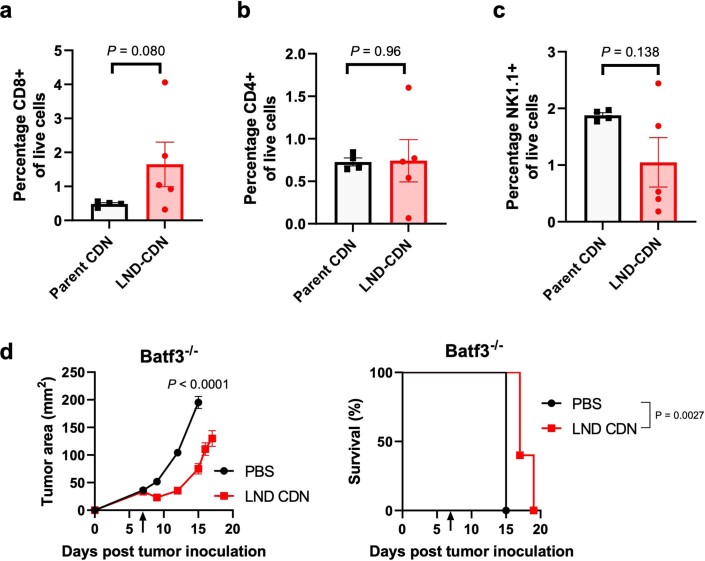
Lymphocyte infiltration in treated tumours and dependence of therapy on *Batf3* + dendritic cells. **a-c**, Mice (*n* = 4 (Parent CDN) or 5 (LND-CDN) animals/group) were inoculated with 5 × 10^5^ MC38 tumour cells and treated with 5 nmol of parent CDN or LND-CDN on day 7. Six days later, tumour-infiltrating CD8^+^ T cells (**a**), CD4^+^ T cells (**b**), and NK cells (**c**) were assessed by flow cytometry (mean ± s.e.m.). **d**, *Batf3*^−/−^ mice bearing MC38 flank tumours (*n* = 5 animals/group) were treated as in **a** with LND-CDN or vehicle control and tumour growth (left, showing mean±s.e.m.) and survival (right) were monitored. Statistical comparisons in **a-d** were made using an unpaired, two-tailed Student’s *t*-test; statistical comparisons between survival curves in **d** were performed using a log-rank (Mantel-Cox) test.

Sustained tumour regression triggered by LND-CDNs suggested the induction of an adaptive immune response mediated by T cells. By 6 d post-treatment, there was a trend toward increased CD8 T-cell but not CD4 T-cell or natural kill (NK) cell infiltration in tumours (Extended Data Fig. 9a–c). Depletion of CD8 T cells (but not NK cells) led to a failure of therapy (Fig. 6a,b). Further, LND-CDN therapy in Batf3^−/−^ mice lacking cross-presenting DCs was ineffective (Extended Data Fig. 9d).

**Extended Data Fig. 10:**
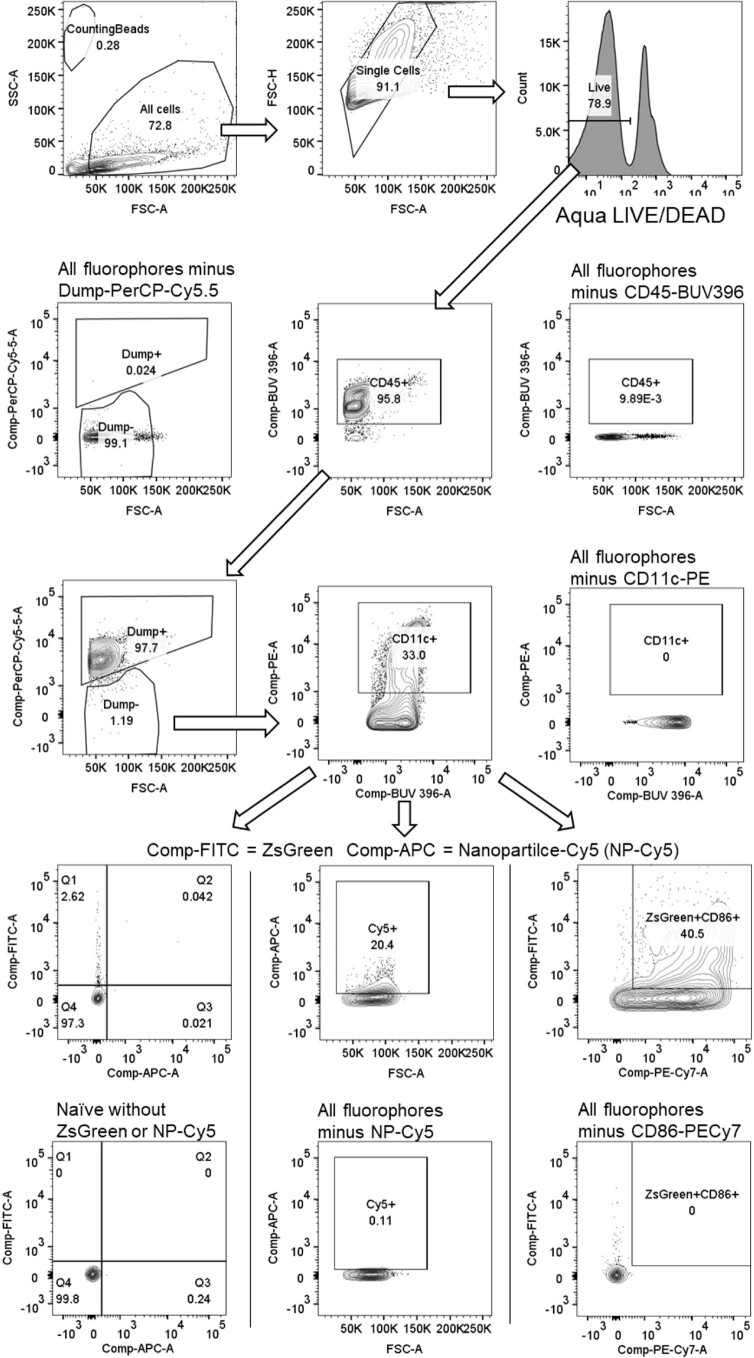
Gating strategy for identifying lymph node dendritic cells containing nanoparticle and tumour antigen. The gating strategy used to identify tumour dendritic cells [CD45^+^ DUMP(Ly6G CD19 CD3e NK1.1)^-^ CD11c^+^] containing tumour antigen (ZsGreen) or nanoparticle (Cy5 detected in the APC channel) is shown (see methods for details on tumour digestion and the antibodies used for staining).

We hypothesized that one mechanism by which LND therapy may be superior to liposome treatment is by virtue of efficient CDN delivery to cancer cells throughout the tumour, ensuring that DCs engulfing tumour cells become fully activated. To test this idea, we employed MC38 tumour cells expressing a stabilized green fluorescent protein (ZsGreen) as a surrogate tumour antigen^33^ (Fig. 6c and Extended Data Fig. 10). Following treatment with Cy5-labelled LND or liposomes, significantly more DCs in tumour-draining lymph nodes (TDLNs) took up LNDs as compared to liposomes, and nearly three times as many DCs were positive for both the CDN carrier and tumour antigen (Ag) following LND-CDN treatment over the first 3 d (Fig. 6d,e). The majority of Ag^+^ DCs were also LND-CDN^+^, whereas Ag^−^ DCs were predominantly LND-CDN^−^ (Fig. 6f), consistent with the idea that many DCs might acquire tumour Ag by taking up LND^+^ dying tumour cells. Enhanced uptake of the CDN nanocarriers was associated with an increased proportion of activated Ag^+^CD86^+^ DCs compared with untreated or liposome-CDN treated tumours (Fig. 6g,h). ELISPOT analysis of splenocytes co-cultured with irradiated tumour cells 14 d post-treatment revealed robust tumour-antigen-specific T-cell responses following LND-CDN therapy (Fig. 6i). Thus, LND-CDN effectively initiates productive priming of T-cell responses in TDLNs.

The analysis above suggested to us that the enhanced efficacy of LND-CDNs was caused by more effective initial distribution of CDNs to cancer cells and/or DCs throughout the tumour bed. To test this idea, we compared LND- versus liposome-CDN administered intratumorally, to alleviate transport barriers for the latter formulation. In this setting, both LND- and liposome-CDNs were highly effective, curing five out of five and four out of five mice, respectively (Fig. 6j). Altogether, these results point to enhanced CDN delivery to tumour cells and DC activation for subsequent T-cell priming as a key factor determining the efficacy of systemic LND-CDN therapy.

## Discussion

STING activation is important for the therapeutic effects of both traditional cancer treatments and many immunotherapies, leading to a great interest in therapeutically targeting STING^5,8,34^. Recent work has made progress on the development of nanocarriers to deliver STING agonists to tumours^12–14,16^, but design goals for optimal systemic delivery of STING agonists remain unclear. Here we show that formulation of CDNs in a nanocarrier promoting efficient tumour penetration led to high levels of therapeutic efficacy following just a single dose of STING agonist, with modest and transient toxicity. Leveraging experience from the field of antibody–drug conjugates^28^, we employed a prodrug approach for linking CDNs to liposomes or LNDs targeting release in endosomes. This approach should ensure effective CDN release both in tumour cells and in dendritic cells, which rely on proteases such as cathepsin S and capthesin L for antigen processing^35,36^. In three different syngeneic tumour models, we found efficacies of LND-CDN treatment ranging from ∼80% tumour rejection in the MC38 model to a ∼50% increase in median survival time in the 4T1 breast cancer model. The MC38 cell line used here was unresponsive to monotherapy with checkpoint blockade, indicating a modest level of immunogenicity, while the 4T1 model is known to be poorly immunogenic and enriched for high levels of immunosuppressive myeloid cells^37^.

Although liposomes and LNDs were equally effective at triggering acute tumour necrosis because both effectively reach the tumour endothelium, our mechanistic studies suggested that efficient delivery of CDN to cancer cells by nanodiscs, and to a lesser degree, dendritic cells, were key differentiating factors. Other studies have demonstrated efficient uptake of nanoparticles in tumour myeloid cells^38,39^. However, dendritic cells, key mediators of T-cell activation, can also be activated by cancer-cell-associated STING agonists in trans following phagocytosis of tumour cells^7,40^. We hypothesize that the ability of LND-CDNs to optimally activate DCs by being in the right place (co-localized with DCs and tumour cells) at the right time (during and following tumour cell death) leads to more effective CD8 T-cell priming and eventual tumour rejection.

PEGylated LND morphologies were first described in the late 1990s^41^, and a few groups have explored this type of LND for drug delivery^42–44^, but the tumour-penetrating capacity of these nanomaterials has not been analysed in detail in vivo. Our computational models of LND versus liposome pore penetration demonstrate that the lack of an enclosed aqueous volume makes nanodiscs amenable to dramatic deformations in response to weak forces, favouring effective convection or diffusion through ECM. These findings also suggest other flexible high-aspect-ratio nanomaterials such as apolipoprotein-based nanodiscs may also be effective for STING agonist delivery.

A potential concern with the use of heavily PEGylated nanocarriers for delivery of an innate immune-stimulatory drug such as CDNs is the potential for the CDN to adjuvant a humoral response against the PEG itself. Anti-PEG antibody responses have been shown to be a source of rapid clearance of PEGylated drugs and liposomal carriers and raise the potential for allergic reactions in patients^29^. This issue has been amplified by the growing awareness of pre-existing anti-PEG responses in humans, and reports of allergic reactions in patients receiving COVID-19 mRNA vaccines that employ PEGylated lipid nanoparticles^45^. Although we did not detect anti-PEG responses on repeat dosing of LND-CDNs, multiple alternative hydrophilic polymers could potentially replace PEG in this system to achieve similar results, including zwitterionic polymers, poly(2-oxazolines) and non-immunogenic hydrophilic polypeptides exhibiting PEG-like behaviour in solution^46^.

## Methods

### Characterization of CDN compounds and intermediates

See Supplementary Methods for detailed methods on synthesis of the parental CDN and intermediate compounds. NMR spectra were recorded in the solvent reported on a 400 MHz Bruker spectrometer using residual solvent peaks as the reference. High-resolution mass spectra were acquired using an Agilent 1260 Infinity II Bio-inert Multisampler, Bio-inert Column compartment, Bio-inert Quaternary Pump, DAD multiwavelength detector and an Agilent 6545 LC-QTOF mass spectrometer, with an Osaka Soda Capcell PAK C1 UG120 (5 µm, 2.0 mm internal diameter × 35 mm length) reverse-phase column. Mass spectra were recorded using a Shimadzu LC–MS-2020 with electrospray ionization. Liquid chromatography–mass spectrometry (LC–MS) was performed on an Agilent 1290 Infinity UPLC system connected to an Agilent 6130 mass spectrometer, a Waters Acquity UPLC system connected to a Waters Acquity SQ mass spectrometer, or an Agilent 1100 Series HPLC system connected to a Waters Micromass ZQ mass spectrometer using reverse-phase C18 columns. Various gradients and run times were selected to best characterize the compounds. Mobile phases were based on acetonitrile/water or methanol/water gradients and contained either 0.1% formic acid or 10 mM ammonium acetate. Compound purity was determined by analysis of the diode array ultraviolet trace of an LC−MS spectrum using the following procedure: compounds were dissolved in dimethyl sulfoxide (DMSO), methanol or acetonitrile, and the solutions were analysed using a Hewlett Packard HP1100 or Agilent 1100 Series LC system connected to a Acquity SQ or Micromass ZQ mass spectrometer using reverse-phase C18 columns. One of two gradients was used to elute the compounds: either a formic acid gradient (acetonitrile containing 0–100% of 0.1% formic acid in water) or an ammonium acetate gradient (acetonitrile containing 0–100% of 10 mM ammonium acetate in water).

### CDN-PEG-lipid preparation

The lipid DSPE-PEG_2000_-PDP (Avanti) was converted to DSPE-PEG_2000_-SH by reducing the disulfide bond in the orthopyridyl disulfide (PDP) group. Specifically, 100.0 mg (33.6 μmol) of DSPE-PEG_2000_-PDP (Avanti) was dissolved in 50.0 mM ammonium acetate buffer (pH 5) with 5.0 mM EDTA (6.0 ml total volume) and then 5.0 equiv. of tris(2-carboxyethyl)phosphine (TCEP) (168 μmol, 48 mg) were added. The solution was incubated at 25 °C for 1 h and then transferred to centrifugal dialysis filters (Amicon) with a 3 kDa molecular weight cut-off membrane, and the sample was washed using fresh reaction buffer and three spin steps to remove the released pyridine-2-thione. The flow-through was monitored by reading the absorbance at 343 nm to ensure the pyridine-2-thione was removed. The solution containing DSPE-PEG_2000_-SH was transferred to a glass vial, the volume was adjusted to 5.0 ml total and buffered to pH 7.6 by adding triethanolamine buffer to a final concentration of 100 mM, and then degassed by bubbling with argon. The CDN-linker maleimide^47^ (33.6 μmol, 38.4 mg) was added (67.2 μl of a 0.500 M stock in DMSO) and the reaction was sealed under argon and stirred overnight at 25 °C. The reaction mixture was purified by reverse-phase HPLC using a C8 column and mixtures of acetonitrile and 0.100 M triethylamine acetate buffered water (pH 7), starting with 40% acetonitrile and ramping to a final mixture with 98% acetonitrile. The CDN-PEG-lipid was isolated in 76% yield (102 mg). The identity was confirmed by matrix-assisted laser desorption/ionization–time of flight mass spectrometry where a series of peaks separated by 44 a.m.u. (the repeat unit mass of PEG) were observed between approximately 3,900 and 4,300 *m*/*z*. The three strongest peaks at 4,070, 4,115 and 4,159 correspond within the margin of error of the technique of ±4 a.m.u. to the [M + Na^+^] species with PEG molecular weights of 2,024, 2,068 and 2,112, which have the expected masses of 4,069, 4,113 and 4,157. The purity was confirmed by observation of a single peak on reverse-phase HPLC when monitored at 260 nm.

### LND and liposome formulation

To prepare LND-CDN, a total of 10.0 μmol of lipid (75.0 mol% HSPC (Avanti), 20.0 mol% DSPE-PEG_5000_-OMe (Avanti), 5.0 mol% CDN-PEG-Lipid) was dissolved in 0.400 ml of ethanol and the solution was warmed to 40 °C until it was completely clear. Subsequently, 20.0 μl aliquots of the ethanol solution were added to a vial containing 1.600 ml of PBS buffer, which was also maintained at 40 °C. The ethanol was removed by dialysis using centrifugal dialysis filters (Amicon) with a 30 kDa molecular weight cut-off membrane as per the manufacturer’s instructions until the solution contained less than 0.2% by volume of ethanol. The solution was filtered through a syringe filter with a 0.2 µm Supor membrane. The concentration of CDN conjugate was determined by diluting an aliquot of the PBS stock solution with methanol to a methanol concentration of 95% by volume and then measuring the absorbance at 260 nm using the molar attenuation coefficient of the CDN-linker (30.3 mM^−1^ cm^−1^). Liposome-CDN was prepared in a similar way, but with a different lipid composition (Supplementary Table 1). Following ethanol removal but before sterile filtration, liposomes were subjected to 5 min total probe sonication (10 W) administered in 30 s intervals followed by cooling in an ice bath to prevent heating of the solution. When noted, nanoparticles were fluorescently labelled by including either DSPE-PEG_2000_-sulfo-Cy5 or DSPE-PEG_2000_-IR800cw at a CDN-to-dye ratio of 5:1 at the time of formulation. DSPE-PEG_2000_-sulfo-Cy5 and DSPE-PEG_2000_-IR800cw were prepared by coupling DSPE-PEG_2000_-NH_2_ (Avanti) to disulfo-Cy5-NHS (Tocris) or IR800cw-NHS (Licor).

### Mice

B6 mice (C57BL/6J), BALB/c mice, STING-deficient Goldenticket mice (*Tmem173*^*gt*^, C57BL/6J-*Sting1*^*gt*^/J) and *Batf3*^−/−^ mice (B6.129 S(C)-*Batf3*tm1Kmm/J) were purchased from Jackson Laboratory. Female mice were used in studies when 8–10 weeks old. All solutions for injection were prepared from sterile PBS buffer (pH 7.4) and were sterile filtered through 0.2 µm syringe filters (Pall, Supor PES) before injection. Mice were killed by CO_2_ asphyxiation for tissue collection. All animal work was conducted under the approval of the Massachusetts Institute of Technology (MIT) Division of Comparative Medicine institute Animal Care and Use Committee in accordance with federal, state and local guidelines.

### Cells

MC38 cells were a gift from J. Schlom (National Cancer Institute) and were cultured in DMEM medium (GE Healthcare Life Sciences) supplemented with 10% fetal bovine serum (FBS) and 100 U ml^−1^ of penicillin and streptomycin. The TC-1 cell line, a human papilloma virus E6- and E7-expressing line derived from C57BL/6 lung epithelia, was provided by T. C. Wu (Johns Hopkins University). TC-1 cells were cultured in RPMI 1640 medium (GE Healthcare Life Sciences) supplemented with 10% FBS and 100 U ml^−1^ of penicillin and streptomycin. 4T1 cells were purchased from American Type Culture Collection (ATCC). 4T1-GFP-Luc (4T1-Luc) cells were generated by transfection of the 4T1 cell line with pGreenFire lentiviral vector (System Biosciences). Cells were cultured in RPMI 1640 Medium with 10% FBS, penicillin (100 U ml^−1^) and streptomycin (100 µg ml^−1^). THP-1-Lucia ISG and Raw-Lucia ISG reporter cells were purchased from Invivogen and were cultured following the vendor’s instructions. To generate the MC38-ZsGreen cell line, MC38 cells were transduced with a lentivirus expressing ZsGreen under the control of the EF-1α promoter. Briefly, 500 μl of virus supernatant was mixed with 500 μl of fresh culture medium (DMEM + 10% FBS) and added to MC38 cells for 2 h at 37 °C and 5% CO_2_, after which the transduction mixture was removed and replenished with fresh culture medium. Three days after transduction, ZsGreen^+^ MC38 cells were sorted by flow cytometry to >99% purity and further expanded for downstream experiments. All cells and cell assays were maintained at 37 °C and 5% CO_2_. All cell lines were tested negative for mycoplasma.

### Simulation details

All simulations were performed with Gromacs 2018.3 at 310 K using the martini 2 forcefield with explicit solvent with a 0.02 ps time step^48,49^. The lipids, solvent, wafer and ions were each independently coupled to a velocity rescaling thermostat with a time constant of 1 ps throughout. Systems were equilibrated for 20 ns using a semi-isotropic Berendsen barostat with a 3 ps time constant. After equilibration, the LND or liposome was pulled through the wafer pore using the constant-force method in Gromacs with a force constant of 200 kJ mol^−1^ nm^−1^ without pressure coupling. The pulling vector was defined between the centres-of-mass of the lipids in either the LND or liposome and the wafer in only the *z* direction. During pulling, the wafer was frozen in the *x* and *y* directions using the freezegrps Gromacs command.

LND systems were first built by creating a 40 nm lipid disc using the insane.py script^50^. Liposome systems were built and equilibrated using the CHARMM-GUI server^51^. Lipids in both systems were then PEGylated using custom code to match experimental composition. PEG molecules (5k) were built using the polyply tool provided by the martini developers. A 60 nm × 60 nm porous wafer was built using the ASE package in python^52^. The overall simulation cell for both the LND and liposome system was 60 nm × 60 nm – 68 nm. Counter-ions and 18% anti-freeze molecules were added to the solvent to avoid water freezing on the rigid substrate.

### In vitro STING activation

STING activation in mouse RAW-Lucia ISG (Invivogen) was performed according to the manufacturer's suggested protocol. STING activation in human THP-1 cells was assessed following methods from a previous report^8^. THP-1 cells were seeded at 1 × 10^5^ cells per well and activated with PMA (80 µM) plus ionomycin (1.3 µM) (eBioscience cell stimulation cocktail) for 2 d. After activation, LND-CDN was added to the cells at titrated concentrations. Cells were cultured with LND for 3 h, washed into fresh media then cultured further for a total of 24 h. Subsequently, culture supernatant was collected and mixed with detection reagent QUANTI-Luc (Invivogen), and secreted Lucia luciferase activity was measured on a Molecular Devices FlexStation3 Multi-mode microplate reader following the manufacturer’s instructions.

### Tumour spheroid formation and imaging

Tumour spheroids were formed by seeding 10,000 MC38 cells per well in a 96 well round-bottom ultra-low attachment plate (Corning) and used after 5 d of growth. Tumoroids were incubated in complete cell media (DMEM with 10% FBS) with sulfo-Cy5-labelled LND-CDN or liposome-CDN at a concentration of 5.0 μM CDN and 1.0 μM dye for 24 h. Following incubation, tumoroids were washed twice with complete media, followed by a PBS buffer wash, and then fixed using 4% paraformaldehyde in PBS for 20 min at 4 °C. Glass slides were prepared for mounting samples by adding two layers of double-sided tape strips (∼5 mm wide) along the edges of the slide to prevent flattening of the tumoroids when the coverslip was added. Tumoroids were washed with PBS and then transferred to glass slides, and excess PBS was blotted away, Vectashield vibrance antifade mounting medium was added, and a coverslip was added. Samples were imaged on a Leica SP8 spectral confocal microscope and analysed using ImageJ.

### Whole-mouse cryofluorescence tomography imaging

C57Bl/6 mice bearing MC38 flank tumours inoculated 10 d previously were intravenously injected via the tail vein with PBS vehicle, LND-CDN (5.0 nmol CDN per mouse), or liposome-CDN (5.0 nmol CDN per mouse) (*n* = 4 per group). LND-CDN and liposome-CDN were both labelled with IR800cw (1.0 nmol per mouse). Mice were treated with equivalent amounts of dye on a molar basis and solutions of nanoparticles displayed equivalent fluorescence as measured by a plate reader before injection. Mice were killed after 4 h and immediately frozen by immersion in hexane cooled with dry ice. Mice were maintained below −80 °C until they were processed by EMIT Imaging using the Xerra system. Imaging was performed using 50.0 μM sections. For each section, in addition to a white-light image, a fluorescence image was acquired with laser excitation at 780 nm and an 835 nm emission filter with an optimized exposure time. Images were processed using ImageJ. To quantify tumour uptake, a region of interest was manually drawn around the tumour using the white-light image on the section corresponding to the coronal centre of the tumour and average fluorescence intensity per pixel was measured. Additional regions of interest were draw in sections 1.0 mm ventral and 1.0 mm dorsal to the centre section, and the average value of the three sections was used. Fluorescence intensities were normalized based on exposure times.

### Tumour and organ histology

B6 mice bearing MC38 flank tumours inoculated 7 d previously were intravenously administered LND or liposome both labelled with 2.0 nmol sulfo-Cy5 dye per mouse via retro-orbital injection, or left untreated (n = 3 per group). Mice were administered equivalent amounts of dye on a molar basis and solutions of nanoparticles displayed equivalent fluorescence as measured by a plate reader before injection. After 24 h, mice were administered 0.30 mg per mouse of fluorescein-labelled, anionic, fixable dextran (relative molecular mass, 2,000,000; ThermoFisher) via retro-orbital injection to label tumour blood vessels, and the mice were killed after 10 min. Tumours were excised and fixed with 4% paraformaldehyde in PBS buffer for 16 h at 4 °C. Subsequently, tumours were embedded in 2.5% agarose and sliced into 100 μm sections using a vibratome (Leica VT1000S). Sections were mounted under a glass coverslip using Vectashield vibrance antifade mounting medium on positively charged glass slides and then imaged using a Leica SP8 spectral confocal microscope using the same laser intensities and gain settings for all samples. Images were analysed in ImageJ. Patent blood vessels were identified using the fluorescein signal with a vascular mask by setting a fixed value threshold above background. The vascular mask was adjusted with the ImageJ functions remove outliers, dilate and fill holes, using the same settings for each image. The vascular mask was then subtracted from a region of interest defined by the tumour margin (dotted white line) to produce an extravascular region of interest, from which the average fluorescence intensity of the nanoparticle signal was measured. To quantify the percentage of extravascular area containing the nanoparticle signal, the extravascular region of interest was set at a constant threshold above background, made binary and the percentage of pixels with non-zero signal was measured. Each point represents the average of two unique tumour sections.

For liver and spleen histology, organs were harvested at 48 h after dosing with LND-CDN or liposome-CDN, then fixed in 4% paraformaldehyde for 24 h. Tissues were transferred into 70% ethanol, embedded in paraffin and stained with haematoxylin and eosin.

### Pharmacokinetics

C57Bl/6 mice were intravenously administered cGAMP-sulfo-Cy5 (1.0 nmol dye per mouse; BioLog, c[3′-[sCya5]-AHC-G(2′,5′)pA(3′,5′)p]), LND-CDN or liposome-CDN, with both nanoparticles at a 5.0 nmol per mouse CDN dose and labelled with 1.0 nmol per mouse sulfo-Cy5 dye (*n* = 9 per group). At predefined time points following injection, groups of three mice were bled retro-orbitally to collect 50 µl of blood. The plasma fraction was collected after centrifugation, diluted 5-fold with PBS buffer containing 5 mM EDTA, and the fluorescence was quantified using a plate reader. Sample concentration was determined against a standard curve prepared in PBS buffer containing 5 mM EDTA and 20% by volume naive mouse plasma. Data are presented as percent injected dose (% ID) and plasma collected at 1 m was taken to represent the maximum injected dose (100% ID). Curves were fit using nonlinear regression and a two-phase decay on GraphPad Prism software.

### Biodistribution

B6 mice bearing MC38 flank tumours inoculated 10 d previously were intravenously administered via retro-orbital injection cGAMP-sulfo-Cy5 (2.0 nmol dye per mouse), LND-CDN (5.0 nmol CDN per mouse, 2.0 nmol dye per mouse) or liposome-CDN (5.0 nmol CDN per mouse, 2.0 nmol dye per mouse) (*n* = 4 per group). Mice were killed after 24 h and tissues of interest were removed. Tissues were weighed and mechanically dissociated until homogeneous in lysis buffer (100 mM HEPES, pH 7.0, 2 wt% Triton-X, 5 mM EDTA) using disposable tissue grinder tubes (Kimble Biomasher). Subsequently, tubes were vortexed for 1 min, centrifuged at 300*g* for 2 min, and the supernatants were transferred to a black 96 well plate for quantification using a fluorescence plate reader (excitation, 640 nm; emission, 680 nm). The concentration of fluorophore was determined using a tissue-specific standard curve prepared with tissue digests from untreated mice. Organ uptake was reported as the percentage of injected dose per gram of tissue.

### Tumour therapy studies

MC38 (5 × 10^5^ cells) or TC-1 (3 × 10^5^ cells) were subcutaneously administered in 100 μl sterile PBS on the right flank of shaved mice. Mice were treated on day 7 or 10 after tumour inoculation. For MC38 rechallenge experiments, mice were inoculated with MC38 (5 × 10^5^ cells) on the flank opposite the initial tumour injection. To initiate orthotopic tumour growth, 5 × 10^5^ 4T1-Luc tumour cells were inoculated into the fourth mammary fat-pad of BALB/c mice and tumours grew for 7 d before administering therapy. For all studies, the mice were randomized into groups before treatment initiation. Tumour size was measured using calipers, and the tumour volume was calculated with the formula *V* = (length × width^2^)/2, where length is the longest dimension and width is the perpendicular dimension. Mice were killed when the tumour volume exceeded 750 mm^3^ or tumour ulceration became severe.

### Cytokine neutralization

Cytokine signalling was neutralized using monoclonal antibodies against IFN-γ (clone XMG1.2, BioXCell), TNF-α (clone XT3.11, BioXCell) or IFNAR-1 (clone MAR1-5A3). Antibodies were administered by intraperitoneal injection on days 6 and 7 (0.200 mg of each antibody per mouse) after tumour inoculation with LND-CDN (5.0 nmol per mouse) administered on day 7.

### Bead-based ELISA cytokine quantification

Tumour and lymph node lysates were analysed using the Legendplex mouse antivirus response panel (Biolegend) following the manufacturer’s suggested protocol and analysed using the LEGENDplex Data Analysis Software Suite. Four hours after treatment, tumours and the tumour-draining inguinal lymph nodes were collected from B6 mice bearing MC38 tumours (day 10 after inoculation) and weighed. Tissues were transferred to disposable tissue grinder tubes (Kimble Biomasher) and lysis buffer (0.1× PBS, 20 mM HEPES pH 7.0, 1 wt% Triton-X, HALT Protease Cocktail (ThermoFisher), 5 mM EDTA) was added (100 μl per lymph node, 2 μl per mg of tumour). The tissues were homogenized and then analysed or flash frozen and stored at −80 °C until later analysis.

### ELISA for anti-PEG antibodies

MaxiSorp ELISA plates were coated with streptavidin at 1 µg ml^−1^ in PBS for 4 h at 25 °C, blocked with PBS + 2% BSA for 18 h at 4 °C, then washed three times with buffer. Biotin-PEG-OH (Creative PEGWorks, catalogue number PJK-1946) was added to the plates in blocking buffer (1 µg ml^−1^) for 2 h at 25 °C. After washing three times with buffer, serum samples and mouse anti-PEG IgG standard antibody (AffinityImmuno kit catalogue number EL-141-PEG-mIGG, starting at 1 µg ml^−1^ followed by two serial dilutions) were added for 2 h prior to washing. Anti-mouse IgG-HRP diluted 1:5,000 in blocking buffer was used as a detection antibody following the manufacturer’s instructions, and absorbance was measured at 450 nm.

### Cell depletions

Cellular subsets were depleted by administering 0.200 mg of depleting antibody by intraperitoneal injection on days 6, 8, 11 and 15 following tumour inoculation. For CD8 T-cell depletion, αCD8a (clone 2.43, rat IgG2b, BioXCell) was compared to an isotype control antibody (clone LTF-2, rat IgG2b, BioXCell). For NK cell depletion, αNK1.1 (clone PK136, rat IgG2a) was compared to an isotype control antibody (clone C1.18.4, rat IgG2a, BioXCell).

### Flow cytometry

Cells were stained with LIVE/DEAD Fixable Aqua (Life Technologies) as per the manufacturer’s instructions and only live cells were analysed. All antibodies are from Biolegend unless otherwise noted. Flow cytometry data were collected using BD FACSDiva 6.1.3 software and analysed using FlowJo v.10.

### Enzyme-linked immunospot assay

Target MC38 cells were treated with 50.0 U ml^−1^ mouse IFN-γ (Peprotech) for 16 h, then irradiated (120 Gy). Effector cells were splenocytes isolated from MC38-tumour-bearing mice 14 d after treatment with LND-CDN or liposome-CDN. A mouse IFN-γ ELISPOT Kit (BD) was used. Targets cells were seeded at 25,000 cells per well. Effector cells were seeded at 500,000 and 250,000 splenocytes per well. Plates were wrapped in foil and cultured for 24 h, then developed according to the manufacturer’s protocol. Plates were scanned using a CTL-ImmunoSpot Plate Reader, and the data were analysed using CTL-ImmunoSpot Software.

### Toxicity analyses

LND-CDN or Lipo-CDN (5 µmol) were administered through intravenous retro-orbital injection into naive C57Bl/6 mice. Blood was collected at 4, 24, 48 and 96 h in Z-gel collection tubes (Sarstedt) and centrifuged to isolate serum. Serum was stored frozen at −80 °C for further analysis. Serum cytokines were analysed using a 13-plex bead-based ELISA cytokine panel (Biolegend LEgendplex), and read by flow cytometry according to the manufacturer’s protocols. Likewise, collected serum was analysed by kits for alanine aminotransferase (ALT), aspartate aminotransferase (AST) and blood urea nitrogen (BUN) as recommended by the manufacturer.

### Statistical analysis

All statistical analyses were performed with GraphPad Prism 9 software. Statistical data are presented as the mean ± s.e.m. unless otherwise noted. Details of the statistical test performed, *P* values and number of replicates are reported in the figure legends. A threshold for significance of *P* < 0.05 was used for all experiments.

### Reporting summary

Further information on research design is available in the Nature Research Reporting Summary linked to this article.

## Online content

Any methods, additional references, Nature Research reporting summaries, source data, extended data, supplementary information, acknowledgements, peer review information; details of author contributions and competing interests; and statements of data and code availability are available at 10.1038/s41563-022-01251-z.

## Supplementary information

Supplementary Information Supplementary Modelling Discussion, Methods, Table 1, video captions.

Reporting Summary

Supplementary Video 1 Supplementary video 1: Animation of a representative coarse-grained simulation of 40 nm diam. PEGylated lipid nanodisc being pulled through a 20 nm diam. pore.

Supplementary Video 2 Supplementary video 2: Animation of a representative coarse-grained simulation of 40 nm diam. PEGylated liposome being pulled through a 20 nm diam. pore.

Supplementary Video 3 Supplementary video 3: Animation of a representative coarse-grained simulation of 40 nm diam. bare liposome being pulled through a 20 nm diam. pore.

Supplementary Video 4 Supplementary video 4: Shown are rotated views of whole-animal reconstructions of untreated tumour-bearing animals or animals dosed with fluorescent LND-CDN 4 hr post administration.

Supplementary Video 5 Supplementary Video 5. 3D reconstruction of whole-animal cryofluroescence tomography imaging of fluorescent liposome-CDN. Shown are rotated views of whole-animal reconstructions of untreated tumour-bearing animals or animals dosed with fluorescent liposome-CDN 4 hr post administration.

Supplementary Data Source data for all main figures and Extended data figures

## Data Availability

The main data supporting the findings of this study are available within the paper and its Supplementary Information files. The associated raw data are available from the corresponding author on reasonable request.

## Code availability

Custom code written for the computational modelling is available from the authors upon reasonable request.
