# Supplementary material for: STING agonist delivery by tumour-penetrating PEG-lipid nanodiscs primes robust anticancer immunity
Source: Nat Mater. 2022 May 23;21(6):710–20. doi: 10.1038/s41563-022-01251-z (PMC9156412; doi:10.1038/s41563-022-01251-z)
Supplement: Supplementary file 1 — Supplementary Modelling Discussion, Methods, Table 1, video captions. [file 41563_2022_1251_MOESM1_ESM.pdf]

nature portfolio

https://doi.org/10.1038/s41563-022-01251-z

Supplementary information

STING agonist delivery by tumour-penetrating PEG-lipid nanodiscs primes robust anticancer immunity

In the format provided by the
authors and unedited

Supplementary Information for:

STING agonist delivery by tumor-penetrating PEG-lipid nanodiscs primes robust anti-cancer immunity

Eric L. Dane[1], Alexis Belessiotis-Richards[2,3,4], Coralie Backlund[1], Jianing Wang[5], Kousuke Hidaka[6], Lauren E. Milling[1,7], Sachin Bhagchandani[1,8], Mariane B. Melo[1], Shengwei Wu[1], Na Li[1], Nathan Donahue[1], Kaiyuan Ni[1], Leyuan Ma[1], Masanori Okaniwa[9], Molly M. Stevens[2,3,4], Alfredo Alexander-Katz[10], and Darrell J. Irvine*[1,7, 10,11,12]

[1]Koch Institute for Integrative Cancer Research, Massachusetts Institute of Technology, Cambridge, Massachusetts, USA
[2]Department of Materials, Imperial College London, London SW7 2AZ, United Kingdom
[3]Department of Bioengineering, Imperial College London, London SW7 2AZ, United Kingdom
[4]Institute of Biomedical Engineering, Imperial College London, London SW7 2AZ, United Kingdom
[5]Millennium Pharmaceuticals, Inc., a wholly owned subsidiary of Takeda Pharmaceutical Company Limited, Cambridge, Massachusetts, USA
[6]Immunology Unit, Takeda Pharmaceutical Company Limited, 26-1, Muraoka-Higashi 2-chome, Fujisawa, Kanagawa 251-8555, Japan
[7]Department of Biological Engineering, Massachusetts Institute of Technology, Cambridge, Massachusetts, USA
[8]Department of Chemical Engineering, Massachusetts Institute of Technology, Cambridge, Massachusetts, USA
[9]Oncology Drug Discovery Unit, Takeda Pharmaceuticals International Co., 40 Landsdowne St., Cambridge, Massachusetts, United States.
[10]Department of Materials Science & Engineering, Massachusetts Institute of Technology, Cambridge, Massachusetts, USA
[11]Ragon Institute of Massachusetts General Hospital, Massachusetts Institute of Technology and Harvard University, USA
[12]Howard Hughes Medical Institute, Chevy Chase, Maryland, USA

Supplementary Discussion – Modeling transport of LND vs. liposomes through small pores

To gain insight into potential differences in transport behavior of LNDs versus liposomes, we performed coarse-grain molecular dynamics simulations on a model LND (diameter = 40 nm) being pulled through a small pore, compared to a model PEGylated or "bare" liposome of similar lipid composition and of the same diameter (**Fig. 2a-b**). Note that while we were unable to experimentally prepare stable CDN-liposomes of this small size, we chose in the model to compare these two nanostructures under the ideal condition of comparing equivalent diameters. We modeled LND/liposome interactions with a rigid pore 20 nm in diameter, a size chosen based on combined experimental and theoretical models predicting that high-density collagen gels representative of tumor ECM have a mean pore size of 20 nm[1] and dextran diffusion data in tumor ECM are well fit by models assuming a matrix porosity of ~16 nm[2]. Adherens and tight junctions between epithelial tumor cells may create barriers of a similar size scale[3]. Even under a modest pulling force of ~330 pN (200 kJ mol^{-1} nm^{-1}), the LND was able to deform and enter a pore smaller than its equilibrium diameter, whereas the liposome was unable to deform sufficiently to enter the pore (**Fig. 2a-c, Supplementary Fig. 4a-b** and **Supplementary Videos 1-3**). To establish the important differences between LNDs and liposomes we developed an analytical model that captures the essential elements of LND translocation through pores. The model considers a LND of radius R_D passing through a circular hole of dimension R_H. The free energy of translocation as a function of the degree of translocation h can be evaluated by the change in line tension of this system comparing the undeformed disc with the constraint of equal overall area. Assuming a small orifice compared to the size of the disc, the free energy in this case scales as equation (1):

$$\Delta G(h) \sim 2\gamma h\left(1-\frac{R_H}{R_D}\right) \quad (1)$$

where γ is the line tension coefficient. The overall barrier can be computed from this same model and has the form $\Delta G_{barrier} \sim 2\pi\gamma R_H(2\alpha^2-(\alpha+1))$, where $\alpha = R_D/R_H$. However, this is not the important quantity when it comes to translocation. Instead, it is the force necessary to overcome this energy barrier. From above, the translocation force scales as $F_T \sim 2\gamma\left(1-\frac{R_H}{R_D}\right)$. The line tension parameter for DOPC lipids has been measured using pore closing dynamics[4,5] to be 7 ± 1 pN. In the presence of molecules that can reduce the line tension, such as Tween 20, the line tension has been measured as low as 0.2 pN. The line tension with the PEG lipids employed here has not been measured, but we expect it to be between bare DOPC and DOPC with Tween 20. This implies that the force would be on the order of ~1 pN. Such forces are on the lower side of typical biological forces[6], and thus we expect that the constant rearranging of the interstitial volume due to cell motions will lead to translocation of LNDs through pores that are smaller than their size. Interstitial flow in tumors has been measured to be in the range of 0.5-50 µm/s[7–9]. The pressure drop necessary for speeds of 10 µm/s moving through local constrictions on the order of 10 nm yields forces on the order of pNs. Such forces would thus be sufficient to drag the LNDs through such constricted apertures in the ECM. More interestingly, in many cases where the size of the disc is slightly larger than the size of the constriction (e.g. between 10-20%), the translocation forces approach thermal forces which are approximated to be on the order of ~0.1 pN for a 30 nm LND, implying such discs might diffuse much more rapidly than harder objects of the same size, even without the need for external forces/flows to drag them through.

Supplementary Methods

Preparation of compound 1 (parent CDN). Step 1: *Preparation of (1R,3R,4R,7S)-3-(6-benzamido-9H-purin-9-yl)-1-((((((2R,3R,4R,5R)-4-((tert-butyldimethylsilyl)oxy)-5-(hydroxymethyl)-2-(2-isobutylamido-6-oxo-1H-purin-9(6H)-yl)tetrahydrofuran-3-yl)oxy)(2-cyanoethoxy)phosphoryl)oxy)methyl)-2,5-dioxabicyclo[2.2.1] heptan-7-yl hydrogen phosphonate.* A mixture of (1S,3R,4R,7S)-3-(6-Benzamido-9H-purin-9-yl)-1-(hydroxymethyl)-2,5-dioxabicyclo[2.2.1]heptan-7-yl hydrogen phosphonate (700 mg, 1.56 mmol) and 5'-O-[bis(4-methoxyphenyl)(phenyl)methyl]-3'-O-[*tert*-butyl(dimethyl)silyl]-2'-O-{(2-cyanoethoxy)[diisopropylamino]phosphanyl}-N-(2-methylpropanoyl) guanosine (2280 mg, 2.35 mmol, 1.5 equiv.) was subjected to azeotropic dehydration with anhydrous acetonitrile, and anhydrous acetonitrile (15 mL) and anhydrous tetrahydrofuran (5 mL) were added thereto. To the mixture was added a mixture of 5-(ethylsulfanyl)-2H-tetrazole (611 mg, 4.69 mmol, 3.0 equiv.) (which was subjected to azeotropic dehydration with anhydrous acetonitrile) and anhydrous acetonitrile (10 mL), and the mixture was stirred overnight under argon atmosphere at room temperature. 70% *tert*-Butyl hydroperoxide (643 µL, 4.69 mmol, 3.0 equiv.) was added thereto, and the mixture was stirred at room temperature for 20 min. To the reaction mixture was added a mixture of sodium thiosulfate (5920 mg, 46.94 mmol, 30 equiv.) and water (3 mL), and the mixture was concentrated under reduced pressure. To the residue was added 80% acetic acid (30 mL), and the mixture was stirred at room temperature for 20 min. The reaction mixture was concentrated under reduced pressure, and the residue was purified by silica gel column chromatography (methanol/ethyl acetate) to give the title compound which was used directly for next step (980 mg, 0.951 mmol, 60.8%). MS: $[M+H]^+$ 1030.2.

Step 2: *Preparation of 2-amino-9-[(5R,7R,8R,12aR,14R,15R,15aS,18R)-14-(6-amino-9H-purin-9-yl)-18-{[tert-butyl(dimethyl)silyl]oxy}-2,10-dihydroxy-10-oxido-2-sulfidohexahydro-14H-15,12a-(epoxymethano)-5,8-methanofuro[3,2-1][1,3,6,9,11,2,10]pentaoxadiphosphacyclotetradecin-7(12H)-yl]-1,9-dihydro-6H-purin-6-one (optical intermediates)* The coupling product from Step 1, (1R,3R,4R,7S)-3-(6-Benzamido-9H-purin-9-yl)-1-((((((2R,3R,4R,5R)-4-((*tert*-butyldimethylsilyl)oxy)-5-(hydroxymethyl)-2-(2-isobutylamido-6-oxo-1H-purin-9(6H)-yl)tetrahydrofuran-3-yl)oxy)(2-cyanoethoxy)phosphoryl) oxy)methyl)-2,5-dioxabicyclo[2.2.1]heptan-7-yl hydrogen phosphonate (980 mg, 0.95 mmol), was subjected to azeotropic dehydration with anhydrous acetonitrile and anhydrous pyridine, and anhydrous pyridine (50 mL) was added thereto. To the mixture was added 2-chloro-5,5-dimethyl-1,3,2-dioxaphosphinane 2-oxide (615 mg, 3.33 mmol, 3.5 equiv.) at room temperature, and the mixture was stirred under argon atmosphere at room temperature for 1 hour. Water (600 µL) and 3H-benzo[c][1,2]dithiol-3-one (240 mg, 1.43 mmol, 1.5 equiv.) were added thereto, and the mixture was stirred at room temperature for additional 30 minutes. To the reaction mixture was added a mixture of sodium thiosulfate (1180 mg, 4.76 mmol, 5.0 equiv.) and water (3 mL), and the mixture was concentrated under reduced pressure. To the residue were added anhydrous acetonitrile (15 mL) and 2-methylpropan-2-amine (5.0 mL), and the mixture was stirred at room temperature for 1.5 hours, and concentrated under reduced pressure. The residue was purified by silica gel column chromatography (methanol/ethyl acetate), and to the obtained residue was added 40% methylamine ethanol solution (30 mL). The mixture was stirred overnight under argon atmosphere at room temperature, and the reaction mixture was concentrated under reduced pressure. The residue was purified by silica gel column chromatography (methanol/ethyl acetate). The obtained residue was resolved into two diastereomers by HPLC (L-column2 ODS, 50x150 mm, mobile

phase as 5 mM aqueous ammonium acetate solution/acetonitrile): tR1 (undesired optical isomer) with shorter retention time (38 mg, 0.047 mmol, 4.35%) and tR2 (desired optical isomer) with longer retention time (322 mg, 0.394 mmol, 36.9%). tR2 was used directly for the next step. MS (tR1): $[M+H]^+$ 817.1. MS (tR2): $[M+14]^+$ 817.1.

Step 3: *2-amino-9-[(5R,7R,8R,12aR,14R,15R,15aS,18R)-14-(6-amino-9H-purin-9-yl)-10,18-dihydroxy-2,10-dioxido-2-sulfanylhexahydro-14H-15,12a-(epoxymethano)-5,8-methanofuro[3,2-l][1,3,6,9,11,2,10]pentaoxadiphosphacyclotetradecin-7(12H)-yl]-1,9-dihydro-6H-purin-6-one sesqui-triethylamine salt (**parent CDN**).* To above tR2 fraction (322 mg, 0.39 mmol) was added methanol (3.0 mL) and triethylamine trihydrofluoride (3.2 mL, 19.71 mmol, 50 equiv.). The reaction mixture was concentrated to remove the methanol, and the residue was stirred at 55°C for 1 hour. The mixture was cooled to room temperature, ethoxy(trimethyl)silane (14 mL, 118.27 mmol, 300 equiv.) was added thereto, and the mixture was stirred at room temperature for 2 hours. The reaction mixture was concentrated under reduced pressure, and the residue was purified by C18 column chromatography (acetonitrile/10 mM triethylammonium acetate buffer solution) to give the final parent CDN (266 mg, 0.294 mmol, 74.6%).MS $[M+H]^+$ 703.1. ^1H NMR (400 MHz, D_2O) δ 1.23 (14H, t, J = 7.3 Hz), 3.15 (10H, q, J = 7.3 Hz), 4.02 (1H, d, J = 8.1 Hz), 4.13-4.24 (2H, m), 4.27-4.42 (4H, m), 4.59 (1H, d, J = 4.4 Hz), 5.01 (1H, s), 5.11 (1H, d, J = 4.2 Hz), 5.61-5.73 (1H, m), 5.95 (1H, d, J = 8.3 Hz), 6.15 (1H, s), 7.87 (1H, s), 8.00 (1H, s), 8.25 (1H, s). ^{31}P NMR (162 MHz, D_2O) δ-1.93, 55.44. ESI HRMS m/z $[M + H]^+$ calcd. For $C_{21}H_{25}N_{10}O_{12}P_2S$ 703.0849, observed 703.0863. Purity > 95 %.

Preparation of compound 2 (CDN prodrug). The synthetic approach is shown in **Supplementary Fig. 1a**. *Preparation of tert-butyl ((S)-1-(((S)-1-((4-(hydroxymethyl)phenyl)amino)-1-oxopropan-2-yl)amino)-1-oxopropan-2-yl)carbamate (**intermediate-1**).* A solution of (*tert*-Butoxycarbonyl)-L-alanyl-L-alanine (2440 mg, 9.09 mmol, Chem-Impex) and 4-aminobenzyl alcohol (1154 mg, 9.09 mmoL, 1.0 equiv.) in DCM (50 mL) was treated with N-ethoxycarbonyl-2-ethoxy-1,2-dihydroquinoline (EEDQ, 2498 mg, 10.0 mmol, 1.1 equiv.) at 25 °C for 40 h. The solid was collected by filtration and washed with DCM (3 x 10mL) and dried with vacuum to obtain the pure product. Additionally, the filtrate was purified by chromatography on silica gel (0-10% MeOH in DCM) to obtain additional pure product. Both batches of product were combined as the **intermediate-1** (2600 mg, 7.12 mmol, 78.3%). LCMS (AA) m/z = 366.2 $[M+H]^+$. ^1H NMR (MeOH-d_4) δ 7.57 (d, J = 8.0 Hz, 2H), 7.30 (d, J = 8.0 Hz, 2H), 4.56 (s, 2H), 4.49-4.45 (m, 1H), 4.09-4.04 (m, 1H), 1.45 (s, 3H), 1.44 (s, 9H), 1.33 (d, J = 8.0 Hz, 3H).

*Preparation of (S)-2-Amino-N-((S)-1-((4-(chloromethyl)phenyl)amino)-1-oxopropan-2-yl)propanamide (**intermediate-2**).* To a round bottom flask charged with **intermediate-1** (700 mg, 1.92 mmol) and acetonitrile (20 mL) was added a solution of hydrogen chloride in dioxane (4M, 25mL, 100 mmol, 52.1 equiv.). The solution was stirred at 25 °C for 90 min. The solvent was removed under vacuum and the remaining residue was suspended in acetonitrile (5 mL) and filtered. The solid was washed with diethyl ether (10 mL x 2) as the **intermediate-2** and used for next step without further purification (730 mg, >100% due to residual HCl/solvent). LCMS (AA) m/z = 284.1 $[M+H]^+$. ^1H NMR (DMSO-*d6*) δ 10.29 (s, br, 1H), 8.74 (s, br, 1H), 8.18 (s, br, 2H), 7.61 (d, J = 8.0 Hz, 2H), 7.37 (d, J = 8.0 Hz, 2H), 4.72 (s, 2H), 4.52-4.47 (m, 1H), 3.90-3.87 (m, 1H), 1.38-1.35 (m, 6H).

*Preparation of N-((S)-1-(((S)-1-((4-(Chloromethyl)phenyl)amino)-1-oxopropan-2-yl)amino)-1-oxopropan-2-yl)-6-(2,5-dioxo-2,5-dihydro-1H-pyrrol-1-yl)hexanamide (**intermediate-3**).* Crude **intermediate-2** (569.9 mg, 1.78 mmol) was suspended in DMF (6 mL) and treated with DIPEA (0.93 mL, 5.34 mmol, 3.0 equiv.). To the above mixture was added a solution of N-succinimidyl 6-maleimidohexanoate (657 mg, 2.13 mmol, 1.20 equiv.) in DMF (4 mL) and kept at 25 °C for 30 minutes. The reaction mixture was then poured into ice-cold buffer (PBS, 100 mL, pH 7.4). The precipitate was collected by centrifugation and washed with water (60 mL x 3), diethyl ether (5 mL x 2) and EtOAc (2 mL) and dried to get **intermediate-3** (560 mg, 1.17 mmol, 66%). LCMS (AA) m/z = 477.2 $[M+H]^+$. ^1H NMR (DMSO-*d6*) δ 9.93 (s, 1H), 8.07 (d, J = 8.0 Hz, 1H), 7.99 (d, J = 8.0 Hz, 1H), 7.62 (d, J = 8.0 Hz, 2H), 7.36 (d, J = 8.0 Hz, 2H), 7.00 (s, 2H), 4.71 (s, 2H), 4.39-4.37 (m, 1H), 4.25-4.24 (m, 1H), 3.38-3.34 (m, 2H), 2.10 (t, J = 8.0 Hz, 2H), 1.52-1.43 (m, 4H), 1.30 (d, J = 8.0 Hz, 3H), 1.22-1.15 (m, 5H).

*Preparation of N-[(2S)-1-{[(2S)-1-{[4-({[(2S,5R,7R,8R,12aR,14R,15R,15aS,18R)-7-(2-amino-6-oxo-1,6-dihydro-9H-purin-9-yl)-14-(6-amino-9H-purin-9-yl)-10,18-dihydroxy-2,10-dioxidohexahydro-14H-15,12a-(epoxymethano)-5,8-methanofuro[3,2-l][1,3,6,9,11,2,10]pentaoxadiphosphacyclotetradecin-2(12H)-yl]sulfanyl}methyl)phenyl]amino}-1-oxopropan-2-yl]amino}-1-oxopropan-2-yl]-6-(2,5-dioxo-2,5-dihydro-1H-pyrrol-1-yl)hexanamide or N-[(2S)-1-{[(2S)-1-{[4-({[(2R,5R,7R,8R,12aR,14R,15R,15aS,18R)-7-(2-amino-6-oxo-1,6-dihydro-9H-purin-9-yl)-14-(6-amino-9H-purin-9-yl)-10,18-dihydroxy-2,10-dioxidohexahydro-14H-15,12a-(epoxymethano)-5,8-methanofuro[3,2-l][1,3,6,9,11,2,10]pentaoxadiphosphacyclotetradecin-2(12H)-yl]sulfanyl}methyl)phenyl]amino}-1-oxopropan-2-yl]amino}-1-oxopropan-2-yl]-6-(2,5-dioxo-2,5-dihydro-1H-pyrrol-1-yl)hexanamide (**2**, **CDN prodrug**).* Compound **1** (Parent CDN, 85 mg, 0.094 mmol)] was co-evaporated with anhydrous acetonitrile (1.5 mL x 3) and dissolved in DMF (1 mL). To this solution was added **intermediate-3** (89.6 mg, 0.188 mmol, 2.0 equiv.) followed by sodium iodide (2.82 mg, 0.0188 mmol, 0.2 equiv.). The reaction was sealed and heated at 50 °C for 50 minutes. The mixture was diluted with DMF (1 mL) and directly purified using reverse phase (C18) chromatography (0-30-100 % acetonitrile/water with 10 mM ammonium acetate as modifier). The pure fractions were collected and lyophilized to obtain the CDN prodrug as a white solid as an ammonium salt (45 mg, 0.039 mmol, 42 %). LCMS (AA) m/z = 1143.3 $[M+H]^+$. ^1H NMR (DMSO-d_6/D_2O) δ 8.18 (s, 1H), 7.99 (br, 2H), 7.56 (d, J = 8.0 Hz, 2H), 7.30 (d, J = 8.0 Hz, 2H), 6.88 (s, br, 2H), 6.06 (s, 1H), 5.91 (d, J = 8.0 Hz, 1H), 5.38 (br, s, 1H), 5.10-5.09 (m, J = 8.0 Hz, 1H), 4.93 (s, 1H), 4.81-4.78 (m, 1H), 4.50 (d, J = 4.0 Hz, 1H), 4.32-4.29 (m, 1H), 4.18-3.93 (m, 9H), 3.34 (t, J = 8.0 Hz, 2H), 2.09 (t, J = 8.0 Hz, 2H), 1.50-1.41 (m, 4H), 1.29 (d, J = 8.0 Hz, 3H), 1.20-1.13 (m, 5H). ^{31}P NMR (162 MHz, DMSO-d_6) δ 28.94 (s, 1P), -2.72 (s, 1P). ESI HRMS m/z $[M + H]^+$ calcd. for $C_{44}H_{53}N_{14}O_{17}P_2S$ 1143.2909, observed 1143.2922. Purity > 95 %.

Negative-stained electron microscopy imaging of lipid nanodiscs. Ten uL of LND in buffer (1 mg/mL lipids) were dropped on a 200 mesh copper grid coated with a continuous carbon film and incubated for 60 seconds, followed by removal of excess solution by touching the grid with a kimwipe. Then 10 µL of negative staining solution, phosphotungstic acid, 1% aqueous solution was dropped on the TEM grid and immediately removed by kimwipe, followed by an additional 10 µL of the stain applied to the grid for 30 seconds, followed by kimwipe removal and drying of the grid at room temperature. The prepared grid sample was mounted on a JEOL single tilt holder equipped in the TEM column. The specimen was cooled down by liquid-nitrogen and imaged on a JEOL 2100 FEG microscope using a minimum dose method to avoid sample damage under

the electron beam. The microscope was operated at 200 kV and with a magnification in the ranges of 10,000~60,000 for assessing particle size and distribution. All images were recorded on a Gatan 2kx2k UltraScan CCD camera.

Cryoelectron microscopy of liposomes. Three uL of liposomes (1 mg/mL lipids) in buffer were dropped on a lacey copper grid coated with a continuous carbon film and blotted to remove excess sample without damaging the carbon layer and flash frozen using a Gatan Cryo Plunge III. The grid was mounted on a Gatan 626 single tilt cryo-holder equipped in the TEM column. The specimen and holder tip were cooled by liquid-nitrogen, maintained during transfer into the microscope and subsequent imaging. Imaging was performed on a JEOL 2100 FEG microscope using a minimum electron dose method essential to avoid sample damage under the electron beam. The microscope was operated at 200 kV and with a magnification in the ranges of 10,000~60,000 for assessing particle size and distribution. All images were recorded on a Gatan 2kx2k UltraScan CCD camera.

Mass spectrometry analysis of CDN release in cells. In non-tissue culture treated 6-well plates, 10 million RAW-Lucia ISG cells were incubated at 37°C in 2.0 mL of complete media (DMEM with 10% FBS) with various concentrations of parent CDN or CDN-PEG-lipid for 18 hours. Subsequently, the cells were detached, transferred to a centrifuge tube and pelleted via centrifugation at 600x*g* for 5 minutes. The supernatant was removed by aspiration and the pellet was resuspended in 1.0 mL of PBS buffer with 1-wt% bovine serum albumin and 1.0 M EDTA and pelleted via centrifugation at 600x*g* for 5 minutes. The supernatant was aspirated and the mass of the pellet was recorded. Cell pellets were lysed with 0.1% formic acid in methanol and then analyzed by LC/MS/MS using a Waters Xselect C18 2.1 mm ID x 30 mm column. Samples were eluted using a solvent ramp of 0% to 95% acetonitrile in water with 0.1% formic acid and quantified based on a parent CDN calibration curve.

Diffusion experiments. Diffusion experiments were performed using QuikPrep® Fast Micro-Equilibrium Dialyzers (Harvard Apparatus) with polycarbonate track-etch membranes of 50.0 nm and 200.0 nm pore size. Membranes were passivated by soaking in PBS buffer with 1.0 wt% bovine serum albumin for 1 hour followed by washing with PBS buffer. A solution of nanoparticle labeled with a sulfo-Cy5 fluorophore in PBS buffer was loaded on one side and PBS buffer without nanoparticle was loaded on the opposite side. The chambers were sealed and incubated at 25 °C with shaking (100-200 rpm) for 24 hours. The solutions from each side of the dialyzer were transferred to a black 96-well plate and the fluorescence was quantified using a plate reader. Percent diffusion was calculated as: Percent Diffusion = $F_{Unloaded}$ /(0.5 x ($F_{Unloaded}$ + F_{loaded})) x 100, where F_{loaded} and $F_{unloaded}$ are the fluorescence intensities of the chambers loaded with nanoparticle and loaded with only PBS, respectively. The assay was performed in triplicate.

Confocal imaging of nanoparticle interactions with cancer cells. MC-38 cells were cultured in complete DMEM and plated in 8 well µ-slides (Ibidi) for 24 hours. Cells were treated with 5 µM of either cy5-labeled LND-CDNs or liposome-CDNs for 4 hours, then wash with complete DMEM to remove excess particles. The cells were then stained with CellBright Steady Membrane 550 membrane stain (Biotim), Hoechst, and Lysotracker Green DND-26 (Thermofisher) in complete DMEM for 30 minutes. Excess dye was washed away using complete DMEM and cells were imaged on an Olympus FV1200 laser scanning confocal microscope with a 60X objective. Images were processed using FIJI.

Serum stability of LND-CDN. Stability experiments were performed using QuikPrep® Fast Micro-Equilibrium Dialyzers (Harvard Apparatus) with cellulose acetate membranes with a 5 kDa

molecular cut off. Membranes were passivated by soaking in PBS buffer to remove sodium azide. A 50 µM solution of LND-CDN in PBS with 10% normal mouse serum was loaded on one side and PBS buffer without nanoparticle was loaded on the opposite side. The chambers were sealed and incubated at 37 °C with shaking (100-200 rpm) for the indicated time points. The retentate was measured for bioactivity using THP-1 cells as described above.

Flow cytometry sample preparation.

Cell-level biodistribution of nanoparticles at 24 h. To characterize nanoparticle distribution at a cellular level, B6 mice bearing MC38 flank tumors inoculated 10 d previously were i.v.-administered LND or liposome both labeled with 2.0 nmol sulfo-Cy5 dye per mouse via retro-orbital injection, or left untreated (n = 4 per group). Mice were administered equivalent amounts of dye on a molar basis and solutions of nanoparticles displayed equivalent fluorescence as measured by a plate reader before injection. After 24 h, mice were euthanized and tumors were excised and stored in PBS buffer on ice. Tumors were weighed, cut into small pieces using scissors, and then digested in Hanks Balanced Salt Solution with Ca^{2+} and Mg^{2+} containing 10.0 mg/mL collagenase II (ThermoFisher) and 0.5 mg/ml DNAse I (Roche) for 1 h at 37 °C with gentle shaking. The dissociated tumors were passed through a 70 µm cell strainer using a syringe plunger to force the material through with frequent rinsing with PBS buffer, isolated by centrifugation at 300g, and then stained with two separate panels. Panel A was designed to identify tumor endothelial cells[10] and included antibodies against mouse CD31 (clone 390, BV421), CD45 (clone 30-F11, FITC), and CD146 (clone ME-9F1, PE). Nanoparticle signal was detected in the APC channel. Endothelial cells were defined as $CD45^-$ $CD31^+$ $CD146^+$ and tumor cells were identified as $CD45^-$ $CD31^-$ $CD146^-$. Panel B was deigned to identify myeloid cell subtypes and included antibodies against mouse Ly6G (clone 1A8, BV421, 1:200), CD45 (clone 30-F11, FITC, 1:200), CD19 (clone 1D3/CD19, PerCP/Cyanine5.5, 1:200), CD3e (clone 145-2C11, PerCP/Cyanine5.5, 1:200), NK1.1 (clone PK136, PerCP/Cyanine5.5, 1:200), CD11b (clone M1/70, PE, 1:200), Ly6C (clone HK1.4, PE-Cy7, 1:200), CD11c (clone N418, APC-Fire 750, 1:200). The myeloid subsets of interest, CD11b+ CD11c- and $CD11c^+$ $CD11b^-$, were gated from $CD45^+$ $Ly6G^-$ and $CD45^+$ $Ly6G^-$ $CD19^-$ $CD3e^-$ $NK1.1^-$ cells, respectively.

Tumor cell viability at 24 h post treatment. MC38 tumor-bearing mice were treated with either parent CDN or LND-CDN (both 5 nmol CDN per mouse) and 24 h later tumors were collected and dissociated into single cells suspension as described above for the 24 h biodistribution study. Cells were analyzed using Panel A, as described above.

Tumor immune cell infiltration 6 d after treatment. MC38 tumor-bearing mice were treated with either LND-CDN or liposome-CDN (both 5 nmol CDN per mouse) and 6 d later tumors were collected and dissociated into single cells suspension as described above for the 24 h biodistribution study. Cells were stained with antibodies against CD3e (clone 145-2C11, BV421, 1:200), CD45 (clone 30-F11, FITC), NK1.1 (clone PK136, PerCP/Cyanine5.5), CD8a (clone 53-6.7, PE-Cy7, 1:200), and CD4 (clone RM4-4, APC-Fire750, 1:200). CD8 T cells were defined as CD45+ CD3e+ CD8+. CD4 T cells were defined as CD45+ CD3e+ CD4+. NK cells were defined as CD45+ CD3e- NK1.1+.

Tumor antigen and nanoparticle co-localization in lymph node dendritic cells. To characterize tumor antigen and nanoparticle trafficking and uptake by dendritic cells in the proximal lymph node, B6 mice bearing MC38-ZsGreen flank tumors were i.v.-administered LND-CDN or liposome-CDN (for both, 5 nmol CDN and 1 nmol sulfo-Cy5 dye per mouse) or PBS via retro-

orbital injection (n = 5 per group per time point). Mice were inoculated 10 d before treatment with $5x10^5$ MC38-ZsGreen cells subcutaneously in the flank. Mice were euthanized one, two, and three days after treatment and lymph nodes were collected, mechanically dissociated, and analyzed by flow cytometry. Cells were stained with antibodies against CD45 (clone 30-F11, BUV395, BD, 1:200), IA/IE (clone M5/114.15.2, BV 421, 1:200), CD19 (clone 1D3/CD19, PerCP/Cyanine5.5, 1:200), CD3e (clone 145-2C11, PerCP/Cyanine5.5, 1:200), NK1.1 (clone PK136, PerCP/Cyanine5.5, 1:200), Ly6G (1A8, PerCP/Cyanine5.5, 1:200), CD11c (clone N418, PE, 1:200), CD86 (clone GL-1, PE-Cy7, 1:100), and CD11b (clone M1/70, APC, 1:200). DCs were defined as $CD45^+CD11c^+$ cells that were $Ly6G^-CD19^-CD3e^-NK1.1^-$. Tumor antigen (ZsGreen) signal was detected in the FITC channel and nanoparticle signal was detected in the APC channel.

Supplementary References

1. Ramanujan, S. *et al.* Diffusion and Convection in Collagen Gels: Implications for Transport in the Tumor Interstitium. *Biophys J* 83, 1650–1660 (2002).

2. Jain, R. K. Transport of molecules in the tumor interstitium: a review. *Cancer Research* 47, 3039–3051 (1987).

3. Choi, I.-K., Strauss, R., Richter, M., Yun, C.-O. & Lieber, A. Strategies to increase drug penetration in solid tumors. *Frontiers in oncology* 3, 193 (2013).

4. Karatekin, E. *et al.* Cascades of Transient Pores in Giant Vesicles: Line Tension and Transport. *Biophys J* 84, 1734–1749 (2003).

5. Sandre, O., Moreaux, L. & Brochard-Wyart, F. Dynamics of transient pores in stretched vesicles. *Proc National Acad Sci* 96, 10591–10596 (1999).

6. Roca-Cusachs, P., Conte, V. & Trepat, X. Quantifying forces in cell biology. *Nat Cell Biol* 19, 742–751 (2017).

7. Munson, J. & Shieh, A. Interstitial fluid flow in cancer: implications for disease progression and treatment. *Cancer Management Res* Volume 6, 317–328 (2014).

8. Walker-Samuel, S. *et al.* Investigating low-velocity fluid flow in tumors using convection-MRI. *Cancer Res* 78, canres.1546.2017 (2018).

9. Chary, S. R. & Jain, R. K. Direct measurement of interstitial convection and diffusion of albumin in normal and neoplastic tissues by fluorescence photobleaching. *Proc National Acad Sci* 86, 5385–5389 (1989).

10. Taguchi, K. *et al.* Isolation of tumor endothelial cells from murine cancer. *J Immunol Methods* 464, 105–113 (2019).

Supplementary Table 1. LND-CDN and liposome-CDN compositions

Nanoparticle	Lipid composition (mol%)				Mean number diameter by DLS (nm) / PDI	Mean diameter by electron microscopy (nm) ± S.D.	Zeta potential (mV)
	HSPC	Cholesterol	DSPE-PEG_{5000}-OMe	CDN-PEG-Lipid			
LND-CDN	75	0	20	5	33.7 / 0.12	25.7 ± 10.0	-1.26
Liposome CDN	60	35	4	1	61.6 / 0.20	90.5 ± 44.9	-2.59
