# Supplementary material for: STING agonist delivery by tumour-penetrating PEG-lipid nanodiscs primes robust anticancer immunity
Source: Nat Mater. 2022 May 23;21(6):710–20. doi: 10.1038/s41563-022-01251-z (PMC9156412; doi:10.1038/s41563-022-01251-z)
Supplement: Supplementary file 2 — Reporting Summary [file 41563_2022_1251_MOESM2_ESM.pdf]

nature research

Corresponding author(s): Irvine, Darrell J.

Last updated by author(s): March 14, 2022

Reporting Summary

Nature Research wishes to improve the reproducibility of the work that we publish. This form provides structure for consistency and transparency in reporting. For further information on Nature Research policies, see our Editorial Policies and the Editorial Policy Checklist.

Statistics

For all statistical analyses, confirm that the following items are present in the figure legend, table legend, main text, or Methods section.

n/a	Confirmed	
☐	☒	The exact sample size (n) for each experimental group/condition, given as a discrete number and unit of measurement
☐	☒	A statement on whether measurements were taken from distinct samples or whether the same sample was measured repeatedly
☐	☒	The statistical test(s) used AND whether they are one- or two-sided<br>*Only common tests should be described solely by name; describe more complex techniques in the Methods section.*
☒	☐	A description of all covariates tested
☐	☒	A description of any assumptions or corrections, such as tests of normality and adjustment for multiple comparisons
☒	☐	A full description of the statistical parameters including central tendency (e.g. means) or other basic estimates (e.g. regression coefficient) AND variation (e.g. standard deviation) or associated estimates of uncertainty (e.g. confidence intervals)
☐	☒	For null hypothesis testing, the test statistic (e.g. F, t, r) with confidence intervals, effect sizes, degrees of freedom and P value noted<br>*Give P values as exact values whenever suitable.*
☒	☐	For Bayesian analysis, information on the choice of priors and Markov chain Monte Carlo settings
☒	☐	For hierarchical and complex designs, identification of the appropriate level for tests and full reporting of outcomes
☒	☐	Estimates of effect sizes (e.g. Cohen's d, Pearson's r), indicating how they were calculated

Our web collection on statistics for biologists contains articles on many of the points above.

Software and code

Policy information about availability of computer code

Data collection	Flow cytometry data was collected using BDFACS Diva. Confocal images were collected using Leica acquisition software. Simulations were performed in Gromacs 2018.3.
Data analysis	FlowJo Version 10 was used to analyse flow-cytometry data. ImageJ was used for image analysis. GraphPad prism 9 was used to plot the figures and to conduct statistical analyses. LEGENDplex™ Data Analysis Software Suite was used to analyze bead-based ELISA cytokine quantification data and CTL ImmunoSpot Software was used to analyze enzyme-linked immunospot assays.

For manuscripts utilizing custom algorithms or software that are central to the research but not yet described in published literature, software must be made available to editors and reviewers. We strongly encourage code deposition in a community repository (e.g. GitHub). See the Nature Research guidelines for submitting code & software for further information.

Data

Policy information about availability of data

All manuscripts must include a data availability statement. This statement should provide the following information, where applicable:

- Accession codes, unique identifiers, or web links for publicly available datasets
- A list of figures that have associated raw data
- A description of any restrictions on data availability

The main data supporting the findings of this study are available within the paper and its Supplementary Information files. The associated raw data are available from the corresponding author on reasonable request.

Field-specific reporting

Please select the one below that is the best fit for your research. If you are not sure, read the appropriate sections before making your selection.

☒ Life sciences ☐ Behavioural & social sciences ☐ Ecological, evolutionary & environmental sciences

For a reference copy of the document with all sections, see nature.com/documents/nr-reporting-summary-flat.pdf

Life sciences study design

All studies must disclose on these points even when the disclosure is negative.

Sample size	Tumor therapy studies used n=10 animals/group based on prior work in the lab demonstrating the ability of this group size to detect at least a 20% different in survival outcomes with 80% power.
Data exclusions	Data points were excluded only if 1) faulty injections were noted during experimental execution or 2) in tumor studies if mice were euthanized for reasons unrelated to tumor area (that is, poor body-condition score).
Replication	No studies have been reported that failed upon repeat. Studies that were repeated are noted in figure captions, and include all studies that demonstrate the primary principles reported in the manuscript.
Randomization	In all studies, mice were randomly binned into experimental groups on the day of the first treatment.
Blinding	Because the same individual planned and executed the study, blinding was not performed.

Reporting for specific materials, systems and methods

We require information from authors about some types of materials, experimental systems and methods used in many studies. Here, indicate whether each material, system or method listed is relevant to your study. If you are not sure if a list item applies to your research, read the appropriate section before selecting a response.

Materials & experimental systems

n/a	Involved in the study	
☐	☒	Antibodies
☐	☒	Eukaryotic cell lines
☒	☐	Palaeontology and archaeology
☐	☒	Animals and other organisms
☒	☐	Human research participants
☒	☐	Clinical data
☒	☐	Dual use research of concern

Methods

n/a	Involved in the study	
☒	☐	ChIP-seq
☐	☒	Flow cytometry
☒	☐	MRI-based neuroimaging

Antibodies

Antibodies used	From BioXCell: anti-mouse CD8a (clone 2.43, rat IgG2b cat. BE0004-1), isotype control antibody (clone LTF-2, rat IgG2b, cat.BE0090), anti-mouse NK1.1 (clone PK136, rat IgG2a cat. BP0036), isotype control antibody (clone C1.18.4, rat IgG2a, cat. BE0085), anti-mouse IFN-gamma (clone XMG1.2, cat. BP0055), anti-mouse TNFalpha (clone XT3.11, cat. BE0058), anti-mouse type 1 interferon receptor (IFNAR-1, clone MAR1-5A3, cat. BE0241). From Biolegend: anti-mouse CD31 (clone 390, BV421, cat. 102423, 1:100), anti-mouse CD45 (clone 30-F11, FITC, cat. 103107, 1:200), anti-mouse CD146 (clone ME-9F1, PE, cat. 134701, 1:100), anti-mouse Ly6G (clone 1A8, BV421, cat. 127627, 1:200), anti-mouse CD19 (clone 1D3/CD19, PerCP/Cyanine5.5, cat. 152405, 1:200), anti-mouse CD3e (clone 145-2C11, PerCP/Cyanine5.5, cat. 100311, 1:200), anti-mouse NK1.1 (clone PK136, PerCP/Cyanine5.5, cat. 108727, 1:200), anti-mouse CD11b (clone M1/70, PE, cat. 101207, 1:200), anti-mouse Ly6C (clone HK1.4, PE-Cy7, cat. 127617, 1:200), anti-mouse CD11c (clone N418, APC-Fire 750, cat. 117351, 1:200), anti-mouse CD8a (clone 53-6.7, PE-Cy7, cat. 100721, 1:200), anti-mouse CD4 (clone RM4-4, APC-Fire750, cat. 116019, 1:200), anti-mouse Ly6G (1A8, PerCP/Cyanine5.5, cat. 127615, 1:200), anti-mouse CD11c (clone N418, PE, cat. 117307, 1:200), anti-mouse CD86 (clone GL-1, PE-Cy7, cat. 105013, 1:100), anti-mouse CD11b (clone M1/70, APC, cat. 101211, 1:200), anti-mouse IA/IE (clone M5/114.15.2, BV421, cat. 107631, 1:200). From BD: CD45 (clone 30-F11, BUV395, cat. 564279, 1:200).
Validation	We used commercially available antibody clones that are routinely used for the purposes of the studies reported in the paper. Pilot experiments were performed to ensure appropriate staining in positive controls. Manufacturers released certificates of analysis for each lot used in these studies.

Eukaryotic cell lines

Policy information about cell lines

Cell line source(s)	MC38 cells were a gift from J. Schlom (National Cancer Institute) and TC-1 cells were provided by T. C. Wu (Johns Hopkins University). THP1 and 4T1 cells were purchased from American Type Culture Collection (ATCC). Raw-Lucia ISG reporter cells were

	purchased from Invivogen.
Authentication	None of the cell lines were authenticated.
Mycoplasma contamination	All cell lines tested negative for mycoplasma contamination.
Commonly misidentified lines (See ICLAC register)	No commonly misidentified lines were used in this study.

Animals and other organisms

Policy information about studies involving animals; ARRIVE guidelines recommended for reporting animal research

Laboratory animals	B6 mice (C57BL/6J), BALB/c mice, STING-deficient Goldenticket mice (Tmem173gt, C57BL/6J-Sting1gt/J), and Batf3−/− mice (B6.129S(C)-Batf3tm1Kmm/J) were purchased from Jackson Laboratory. Female mice were used in studies when 8-10 weeks old. Animal facilities are maintained at 70° F; relative humidity is maintained at 30-70%, with a light cycle of 14 hours followed by a dark cycle of 10 hours
Wild animals	The study did not involve wild animals.
Field-collected samples	The study did not involve samples collected from the field.
Ethics oversight	All studies were approved by the MIT Committee on Animal Care (CAC).

Note that full information on the approval of the study protocol must also be provided in the manuscript.

Flow Cytometry

Plots

Confirm that:

☒ The axis labels state the marker and fluorochrome used (e.g. CD4-FITC).

☒ The axis scales are clearly visible. Include numbers along axes only for bottom left plot of group (a 'group' is an analysis of identical markers).

☒ All plots are contour plots with outliers or pseudocolor plots.

☒ A numerical value for number of cells or percentage (with statistics) is provided.

Methodology

Sample preparation	Details of sample preparation are provided in Methods, including tissue-processing steps. Briefly, lymph nodes were mechanically digested and tumors were digested using both enzymes and mechanical dissociation in order to obtain single cell suspensions.
Instrument	BD FACS LSR Fortessa.
Software	FlowJo V10
Cell population abundance	The relative abundance of each gate is shown in Extended data Figures 8 and 10.
Gating strategy	The gating strategies are outlined in Extended data Figures 8 and 10.

☒ Tick this box to confirm that a figure exemplifying the gating strategy is provided in the Supplementary Information.
